# TET1s deficiency exacerbates oscillatory shear flow-induced atherosclerosis

**DOI:** 10.7150/ijbs.69281

**Published:** 2022-02-28

**Authors:** Kai Qu, Caihong Wang, Lu Huang, Xian Qin, Kun Zhang, Yuan Zhong, Qingfeng Ma, Wenhua Yan, Tianhan Li, Qin Peng, Yi Wang, Hans Gregersen, Chaojun Tang, Juhui Qiu, Guixue Wang

**Affiliations:** 1 Key Laboratory for Biorheological Science and Technology of Ministry of Education, State and Local Joint Engineering Laboratory for Vascular Implants, College of Bioengineering Chongqing University, Chongqing, China; 2 Institute of Systems and Physical Biology, Shenzhen Bay Laboratory, Shenzhen, China; 3 GIOME, Department of Surgery, the Chinese University of Hong Kong, Hong Kong, China; 4 Cyrus Tang Hematology Center, Collaborative Innovation Center of Hematology, Soochow University, Suzhou, China

**Keywords:** Atherosclerosis, TET1s, connexin 40 (CX40), oscillatory shear flow (OSS), vascular endothelial barrier

## Abstract

**Background:** TET1 has been implicated in regulating inflammation and cardiovascular disease, but a newly discovered short isoform of TET1 (termed TET1s) exhibits higher expression in adult tissues than full-length TET1. However, the precise role of TET1 in cardiovascular disease remains undefined.

**Methods and Results:** Based on *TET1^-/-^* knockout mice (with deletion of both TET1 and TET1s ) and *TET1^cs/cs^* mice (with deletion of only TET1), we found that TET1s deletion in *TET1^-/-^* mice resulted in more serious atherosclerotic lesions in the whole aorta than TET1cs/cs in the *ApoE^-/-^* background mice fed a high-fat diet. Atherosclerotic lesions with Oil red staining were dramatically localized in the aortic arch, abdominal aorta and ligated LCA, where they were exposed to OSS. Furthermore, the OSS-induced depression of TET1s* in vitro* and *in vivo* increased inflammatory cell and red blood cell infiltration into the subendothelial layer by impairing the vascular intimal barrier. TET1s upregulation enhanced vascular endothelial barrier function by increasing gap protein connexin 40 (CX40) expression as measured by RNA-seq and was confirmed by CX40 knockdown. TET1s interaction with Sin3a increased the global and CX40 promoter histone H3K27 acetylation levels, but not DNA methylation, to induce CX40 expression.

**Conclusions:** These data demonstrate the unexpected discovery that laminar shear stress induces TET1s expression to protect the vascular endothelial barrier by increasing CX40 expression in ECs and that TET1s overexpression may be the core step for OSS-induced atherosclerosis therapy.

## Introduction

Atherosclerosis is a chronic inflammatory disease that results in angina, myocardial infarction, or ischemic stroke, which are the leading causes of morbidity and mortality worldwide^1^,^2^. Vascular endothelial cells (ECs) play a central role during atherosclerosis development from the initial to the advanced stage. Specifically, endothelial barrier dysfunction can be an avenue for lipid accumulation in the subintimal space, erythrocyte leakage and inflammatory cell infiltration, which accelerate atherosclerosis development^3^. Because ECs act as a unique barrier separating the vascular wall from the blood, they are highly responsive to various hemodynamic forces, including shear stress^4^. Laminar shear stress (LSS) improves barrier integrity through the stabilization of cell-cell junctions, while perfusion defects or low shear stress increase vascular permeability 5. Not surprisingly, oscillatory shear stress (OSS) impairs endothelial function, including endothelial barrier function^6^. However, the molecular signaling pathway that regulates the endothelial barrier and can be specifically activated by OSS has still not been identified *in vitro* and *in vivo*.

DNA modifications play an important role in atherosclerosis^7^. DNA demethylation facilitates transcriptional activation^8^. Several studies have shown that OSS controls epigenomic DNA methylation patterns in a DNA methyltransferase-dependent manner both *in vitro* and *in vivo*, which in turn alters endothelial gene expression and induces atherosclerosis^9^,^10^. Tet methylcytosine dioxygenase 1 (TET1), a member of the ten-eleven translocation (TET) enzyme family, regulates gene expression by active DNA demethylation^11^. It can oxidize 5-methylcytosine (5mC) to 5-hydroxymethylcytosine (5hmC), which can be further processed into cytosine through base excision repair, ultimately leading to DNA demethylation^12^. In addition, TET1 can also mediate gene repression by facilitating the binding of the histone deacetylase complex through interaction with Sin3a^13^,^14^. TET1 is an important transcriptional mediator of inflammatory cytokines in macrophages^15^,^16^. TET1 is also enriched in endothelial cells and mediates atherosclerosis development through epigenetic modification^17^. Although TET1 may participate in atherosclerosis development, the precise role of TET1 in OSS-induced atherosclerosis is unclear.

Furthermore, recent studies have reported that a novel short isoform of TET1 (termed TET1s) lacks the N-terminus, including the CXXC domain, a DNA-binding module, and presents different expression and functional patterns from full-length TET1 (termed TET1-FL)^18^,^19^. TET1s is the predominant transcript, with ~10-fold higher expression levels than TET1-FL, in most somatic tissues (e.g., heart, kidney, liver, muscle, spleen and brain)^20^. TET1s is aberrantly activated in multiple cancer cells, suggesting that it may be involved in tumorigenesis^18^. The role of TET1s in the brain has also been investigated, where it was found to modulate hippocampal-dependent memory formation together with TET1-FL^20^. In addition, TET1s can regulate epigenetic memory erasure in embryonic stem cells (ESCs)^19^. Given that TET1s is the predominant transcript of TET1 in somatic tissue and epigenetic modification plays an important role in atherosclerosis development, we postulated that TET1s is the true mediator of this process.

In the present study, we investigated the role of TET1s in atherosclerosis, particularly in vascular endothelial cells using an animal model. We demonstrate that TET1s expression in vascular endothelial cells is the predominant transcript compared with TET1-FL, and OSS dramatically inhibits TET1s expression. We also show that TET1s can significantly delay the development of atherosclerosis by comparing two different knockout mice, *ApoE^-/-^TET1^-/-^* and *ApoE^-/-^TET1^cs/cs^*. The mechanism for this effect is the enhancement of vascular endothelial barrier function by TET1s through an increase in gap junction protein CX40 expression via increased CX40 promoter histone acetylation. Our findings indicate that OSS specificity inhibits TET1s in EC *in vitro* and *in vivo* and implicates TET1s CX40 as a potential target for intervention against atherosclerosis associated with the OSS-induced EC barrier.

## Materials and Methods

### Animals

*TET1^-/-^* mice and *TET1^cs/cs^* mice have been described previously^19^,^21^. *TET1^-/-^*mice lack TET1-FL and TET1s, and *TET1^cs/cs^* mice lack TET-FL. *ApoE^-/-^* mice were obtained from Beijing Vital River Laboratory Animal Technology Co., Ltd. *ApoE^-/-^ TET1^-/-^* and *ApoE^-/-^TET1^cs/cs^* mice were obtained by crossing TET1^-/-^ mice with *ApoE^-/-^* mice and crossing TET1^cs/cs^ mice with *ApoE^-/-^* mice (Table S1).

### Cell culture

Plasma fibronectin (ScienCell)-coated culture vessels (40 μg/mL) were prepared 12 h before subculture. Primary human umbilical vein endothelial cells (p-HUVECs) were purchased from ScienCell Company and cultured in EC medium ECM (ScienCell) supplemented with 5% fetal calf serum (FCS, ScienCell) and 1% penicillin/streptomycin (ScienCell). and endothelial growth supplement (EGS, ScienCell). Thp-1 cells were purchased from ATCC and cultured in Dulbecco's modified Eagle medium (Gibco) supplemented with 10% FCS and 1% penicillin/streptomycin.

All cell types were cultured at 37°C in a 5% CO2 atmosphere and tested negative for mycoplasma.

### Cell transfections

P-HUVECs were transfected at 60-70% confluence with connexin 40 (CX40) CRISPR/Cas9 KO plasmids (h) (sc-401031, Santa Cruz Biotechnology) and CX40 HDR (sc-401031-HDR, Santa Cruz Biotechnology) using UltraCruz® Transfection Reagent (sc-395739, Santa Cruz Biotechnology) according to the manufacturer's protocol. Puromycin was used to select stable knockout (KO) cells.

### Lentiviral constructs

For adenovirus overexpression, the TET1s transcript sequence was cloned into the pAV[Exp]-CMV>EGFP vector (VectorBuilder). Adenovirus particles were generated by transfection of HEK293A cells. Supernatants were collected 48 and 72 h after transfection and concentrated by centrifugation (20000 × g, 4°C, 2 h). P-HUVECs treated with adenovirus for TET1s overexpression or empty vector as a negative control were washed with media 12 h after virus transduction. Experiments were performed 48 h after virus transduction.

### Isolation of mouse endothelial cells

Murine aorta obtained from anesthetized mouse, is perfused with PBS containing 1,000 U/mL of heparin to wash vessel. Remove fat and connecting tissue. The aorta is dissected out from the aortic arch to the thoracic aorta, and immersed in 20% FBS DMEM containing 1,000 U/mL of heparin. Separate the aorta to aortic arch and thoracic aorta. Separate adventitia from aortic arch and thoracic aorta respectively. Adventitia -free aorta immerse in PBS containing 0.25% of trypin at 37°C for 5 minutes. Add 10% FBS to termination and washing intima with PBS to separate the endothelial cells. PBS was centrifuged and endothelial cells were collected. The proportion of endothelial cells was tested by flow cytometry.

### RNA isolation and RT-qPCR

Total RNA was extracted from different treated p-HUVECs or tissues using RNAiso Plus (#9109, Takara Biomedical Technology). RNA was reverse transcribed using the reverse transcription PCR Reagent Kit (RR047A, Takara Biomedical Technology). Then, the real-time quantitative PCR (RTPCR) system (CFX Connect^TM^, Bio-Rad) was used to evaluate the relative mRNA levels. GAPDH served as the control. Gene primers used for qPCR are shown in Table S2.

### Shear stress

The cultured ECs were subjected to shear stress in a parallel-plate flow chamber as described earlier^22^. Cells were exposed to laminar flow at a shear stress of 12 dyn/cm^2^ for 24 h or oscillatory flow at a frequency of 1 Hz and a shear stress of 0.5 ± 4 dyn/cm^2^. Contrary to the laminar flow condition, the oscillatory flow apparatus contained a piston pump with a frequency of 1 Hz.

### Western blot

Total protein was extracted from p-HUVECs or tissues using cold lysis buffer (P0013, Beyotime Biotechnology) Protein concentrations were determined using a BCA Protein Assay Kit (P0010, Beyotime Biotechnology). Equal protein amounts were separated by SDS-polyacrylamide gel electrophoresis and transferred to polyvinylidene difluoride membranes (Millipore). Membranes were blocked in BSA (5% in TBS-T) for 2 h. Primary antibodies (H3K27, 4620S, Cell Signaling Technology; Ac-H3K27, 8173S, Cell Signaling Technology; TET1, GTX124207, GeneTex; Sin3A, 14638-1-AP, Proteintech; CX40, 36-4900, Invitrogen; GAPDH, 10494-1-AP, Proteintech) were diluted in blocking solution and incubated overnight (4°C). GAPDH was used as a loading control. Horseradish peroxidase-conjugated secondary antibodies (Cell Signaling Technology, CST) were incubated for 1 h at RT and detected by the ECL Plus Kit (P0018S, Beyotime Biotechnology). The protein contents were assessed using Labwork image analysis software.

### Quantitative measurements of atherosclerosis

The heart, aorta and carotid artery were removed and fixed in 4% paraformaldehyde. Then, the aorta and carotid artery were cut open longitudinally. The sections of the aorta root and carotid artery were stained with Oil Red O (ORO), and the opened aorta and carotid artery were also stained with ORO to evaluate the extent of atherosclerosis. Atherosclerotic plaque images were analyzed by ImageJ software.

### Immunohistochemistry

The tissues were fixed in 4% paraformaldehyde and then cut into 4 μm sections. The sections were blocked with QuickBlock Reagent (P0260, Beyotime Biotechnology) for 2 h, incubated overnight with primary antibodies (TET1, GTX124207, GeneTex; CD68, GB11067, Servicebio; F4/80, GB11027, Servicebio; CD11b, GB11058, Servicebio; Ly6G, GB11229, Servicebio), incubated with secondary antibody for 30 min and stained with DAB at room temperature. Subsequently, the sections were counterstained with hematoxylin. The intensity of positive staining was analyzed with ImageJ software.

### Immunofluorescence

Sections of arteries, carotid arteries, aortic arches or cells were fixed with 4% paraformaldehyde for 30 min. Then, the samples were washed with PBS and permeabilized/blocked with 0.1% Triton X-100 (in 5% BSA). Subsequently, the samples were incubated with the primary antibody (TET1, GTX124207, GeneTex; VE-cadherin, sc-9989, Santa Cruz Biotechnology) in a wet box at 4°C overnight. After the samples were washed with PBST (PBS with 0.1% Tween-20) 5 times, the samples were incubated with secondary antibodies (Abcam) for 1 h and actin-stain 647 phalloidin (A22287, Invitrogen) for 1 h. Nuclei were labeled with DAPI (Invitrogen) in the dark. The fluorescent signal of sections was detected by SP8 confocal microscopy (Lecia).

### Permeability experiment

Endothelial barrier function* in vivo* was measured by tail vein injection with Evans blue for 45 min. Mice were injected with 5 mg/kg 1% Evans blue (#E2129, Sigma). Then, they were anaesthetized and perfused with normal saline with 1% heparin. Evans blue dye in the aorta, carotid artery and aorta root was photographed via a digital camera (Nikon, Japan), extracted by formamide and quantified using spectrophotometry (Thermo, US) as previously described^23^.

### Transwell assay

Endothelial barrier integrity was analyzed by FITC-dextran (4 kDa, Sigma-Aldrich) through the endothelial monolayer^24^,^25^. Overall, p-HUVECs (48 h post transfection) were seeded into fibronectin-coated cell culture inserts (pore size: 0.4 μm, Corning). When cells reached 100% confluence, 5 mg/mL FITC-dextran was added to the upper compartment for 1 h. FITC-dextran passage was determined by spectrophotometric detection.

### Transendothelial migration assay

p-HUVECs (48 h post transfection) were seeded into fibronectin-coated cell culture inserts (pore size: 8 μm, Corning) with serum-free medium. Thp-1 differentiated into macrophage cells through adding phorbol ester (PMA) for 24. When p-HUVECs reached 100% confluence, macrophages (labeled with hochest) were added to the upper compartment and complete medium was added to chamber to induce transendothelial migration of macrophage. The number of migrated macrophages was determined by inverted fluorescence microscope.

### Immunoprecipitation

Immunoprecipitation has been described previously^26^. In brief, p-HUVECs were lysed using Cell Lysis Buffer for Western blotting or IP (P0013, Beyotime Biotechnology) for 15 min on ice. Cell lysate was incubated with anti-Sin3A antibody for 2 h at 4°C. Then, 50 µl Protein A/G magnetic beads (HY-K0202, MedChemExpress, MCE) were added to the cell lysate sample containing the antigen for 2 hours at 4°C. Beads were washed with wash buffer (25 mM Tris-HCl pH 7.5, 100 mM NaCl, 10 mM MgCl2), and elution buffer was used to elute target antigen. The final solution was used as a sample for denaturing SDS-PAGE.

### ChIP assay

The ChIP assay was performed using the SimpleChIP® Plus Enzymatic Chromatin IP Kit (9005s, CST) according to the manufacturer's instructions, with minor modifications. The cells were cross-linked with 1% formaldehyde for 10 min at room temperature. DNA was broken to yield 150 to 9,00 bp fragments using micrococcal nuclease. Chromatin was then immunoprecipitated using anti-Sin3A (8056S, CST) or anti-AcH3K27 (4353, CST) antibodies. Finally, DNA purification using spin columns and quantification of DNA by PCR were performed (Table S3).

### Dot-blot assay

The dot-blot assay has been described previously^27^. Genomic DNA was extracted from cultured p-HUVECs using a Genomic DNA Purification Kit (K0512, Thermo Scientific™) according to the manufacturer's instructions. The concentration of DNA was quantified using a nucleic acid concentration detector (NanoDrop 2000, Thermo Scientific™). DNA samples loaded on a polyvinylidencefluoride (PVDF) membrane (ISEQ00010, Millipore) using a 96-well dot-blot apparatus (1706545, Bio-Rad). The membrane containing DNA was heated at 80°C for 30 min and blocked with 5% nonfat milk for 1 h at room temperature. Then, the membrane was incubated in a monoclonal anti-5hmC (A-1018-100, Epigentek) antibody and anti-5mC antibody (A-1014-100, Epigentek) at 4°C overnight. The corresponding secondary antibody conjugated with peroxidase was applied to visualize 5hmC and 5mC. The densities of the dots were assessed using Labwork image analysis software.

### Pyrosequencing assays

The Pyrosequencing has been described previously^28^. DNA was modified by bisulfite using the QiagenEpiTect Bisulfite Kit (59104, QIAGEN). Modified DNA was PCR-amplified using a PyroMark PCR Kit (978703, QIAGEN). Each amplicon was sequenced on a pyrosequencer (PyroMark Q96 ID, QIAGEN). The percentage of cytosine methylation within CpG dinucleotides was determined using Pyro Q-AQ software. Amplification and sequencing primers were designed with PyroMark Assay Design 2.0 (Table S4.).

### Study approval and ethics statement

All animal procedures and patients sample study were reviewed and approved by the Animal Care and Ethic Committee of Chongqing University. All animals were housed, cared for, and used in compliance with the guidelines regarding the humane use and care of laboratory animals for biomedical research published by the National Institutes of Health (No. 85-23, revised 1996).

### Statistical analysis

Statistical analyses were performed with Statistical Package for Social Sciences version 23.0. Data were presented as mean ± S.D. Statistical significance between 2 groups was evaluated with Student's t test (unpaired, two-tailed) and statistical significance among multiple groups was analysed using ANOVA followed by Bonferroni's multiple comparison test. All biochemical experiments and representative images were performed in at least three independent experiments.

## Results

### TET1s deficiency promotes atherosclerotic development in ApoE^-/-^ mice

**Figure 1:**
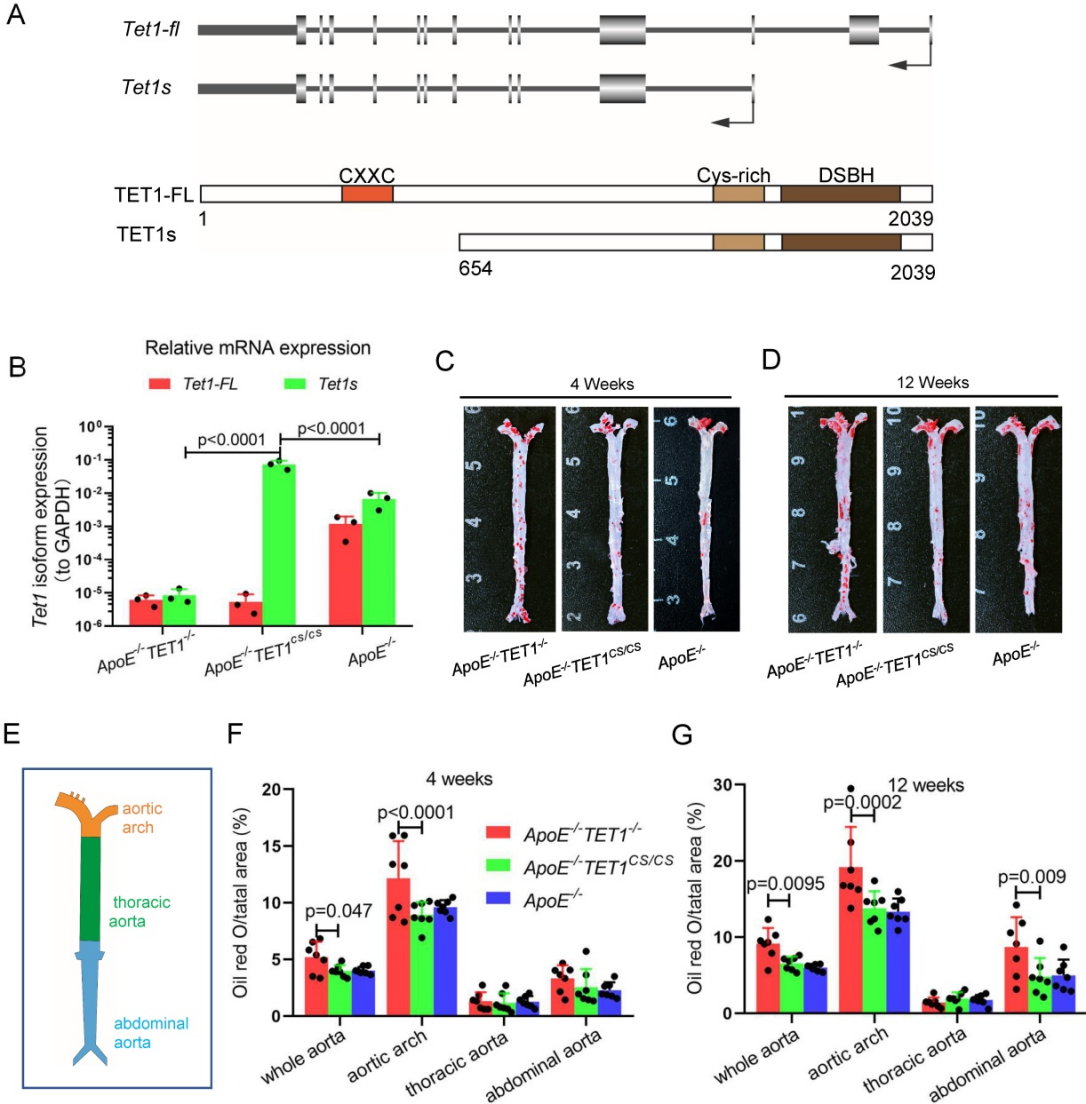
** TET1s deletion exacerbates atherosclerotic lesions in high-fat-diet *ApoE-/-* mice.** (A) Schematic of TET1-FL and TET1s (genes and proteins). (B) RT-qPCR was used to measure the Tet1-FL and Tet1s mRNA levels in aortic ECs from *ApoE^-/-^ TET1^-/-^*, *ApoE^-/-^TET1^cs/cs^* and *ApoE^-/-^* mice (n=3 per group). (C-G) *ApoE^-/-^ TET1^-/-^*, *ApoE^-/-^TET1^cs/cs^* and* ApoE^-/-^* mice (8 weeks old) were fed a high-fat diet for 4 and 12 weeks, respectively. (C-D) The aortic plaques of *ApoE^-/-^ TET1^-/-^* and *ApoE^-/-^TET1^cs/cs^* mice were tested by ORO staining and *en face* microscopy*.* (E) The model of whole aortas divided into aortic arch, thoracic aorta and abdominal aorta. (F-G) The lesion areas in the whole aorta, aortic arch, thoracic aorta, and abdominal aorta sections were analyzed (n>7 per group).

To investigate the function of TET1 in atherosclerosis, we first introduced the structure of the TET1 gene (Figure 1A) and tested the expression of TET1-FL and TET1s in ECs from C57BL/6 mice with whole aortas. Arterial EC TET1-FL and TET1s expression was detected by real-time quantitative polymerase chain reaction (RT-qPCR) and Western blot (WB). TET1s expression in ECs was the predominant transcript compared to TET1-FL with the mRNA levels of TET1s ~7.5-fold higher than TET1-FL and the protein expression ~2.8-fold higher (Figure S1A-C).

**Figure 2:**
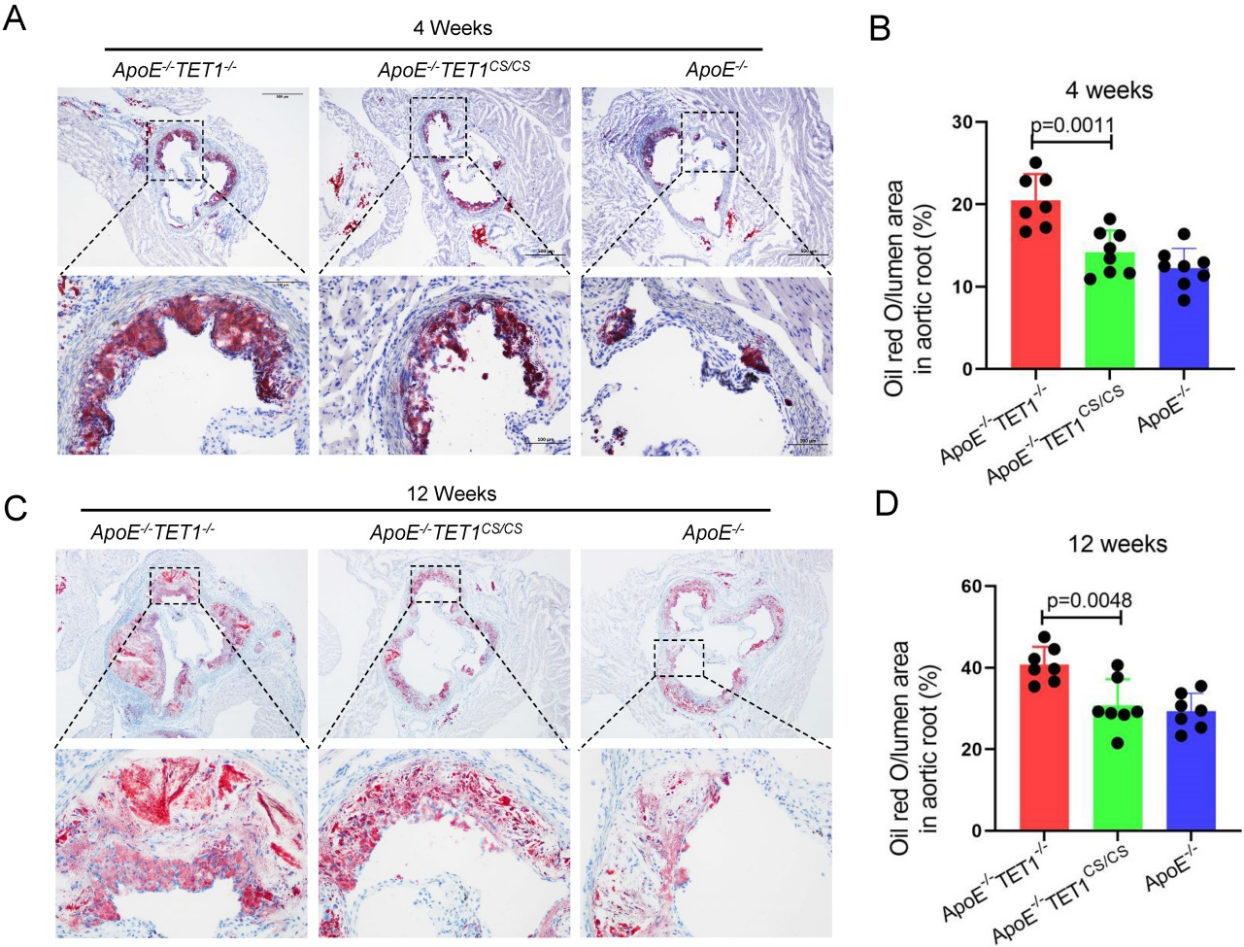
**TET1s deletion increases the plaque area in aortic root.** (A-D) *ApoE^-/-^ TET1^-/-^*, *ApoE^-/-^TET1^cs/cs^* and* ApoE^-/-^* mice (8 weeks old) were fed a high-fat diet for 4 and 12 weeks, respectively. (A, C) Representative photomicrographs of aortic root slice ORO staining. (B, D) Quantitative analysis of atherosclerotic plaque areas in the aortic root (n>7 per group). All data were presented as the mean ± SD.

To investigate the role of TET1s in the development of atherosclerosis, two kinds of double knockout mice, *ApoE^-/-^TET1^-/-^*, *ApoE^-/-^TET1^cs/cs^* mice, were constructed by crossing *TET1^-/-^*mice and *TET1^cs/cs^* mice with *ApoE^-/-^* mice. Their difference was only whether they expressed TET1s, which was confirmed by RT-qPCR (Figure 1B). All *ApoE^-/-^, ApoE^-/-^TET1^-/-^* and *ApoE^-/-^TET1^cs/cs^* mice (8 weeks old) were fed a high-fat diet for 1, 4, and 12 weeks. We found that the area of plaques in *ApoE^-/-^TET1^-/-^* mice was dramatically larger in whole aortas (Figure 1C-D) and aortic roots (Figure 2A-D) relative to *ApoE^-/-^TET1^cs/cs^* and *ApoE^-/-^* mice fed a high-fat diet for 4 and 12 weeks by using ORO staining. However, there was no difference between *ApoE^-/-^*, *ApoE^-/-^TET1^cs/cs^* and* ApoE^-/-^TET1^-/-^* mice fed a high-fat diet for 1 week (Figure S2 A-D). Different vessel regions show a specific hemodynamic pattern, and the aortic arch (AA) and abdominal aorta are exposed in OSS^29^. The size of plaques in *ApoE^-/-^TET1^-/-^* mice was dramatically larger in the aortic arch, and abdominal aorta relative to *ApoE^-/-^TET1^cs/cs^* and *ApoE^-/-^* mice fed a high-fat diet for 4, 12 weeks (Figure 1F-G). We also found plasma lipids levels and hepatic slice oil red O staining were no difference between* ApoE^-/-^TET1^cs/cs^* mice and* ApoE^-/-^TET1^-/-^*mice fed a high-fat diet for 12 weeks (Figure S3). This shows the effect of TET1s deficiency promoting atherosclerotic development is not through regulation of plasma lipids.

Together, these results demonstrate that deletion of TET1s significantly promotes the development of atherosclerosis and that the effect may be involved in hemodynamic stimuli.

### TET1s deficiency exacerbates disturbed flow-induced atherosclerosis

**Figure 3:**
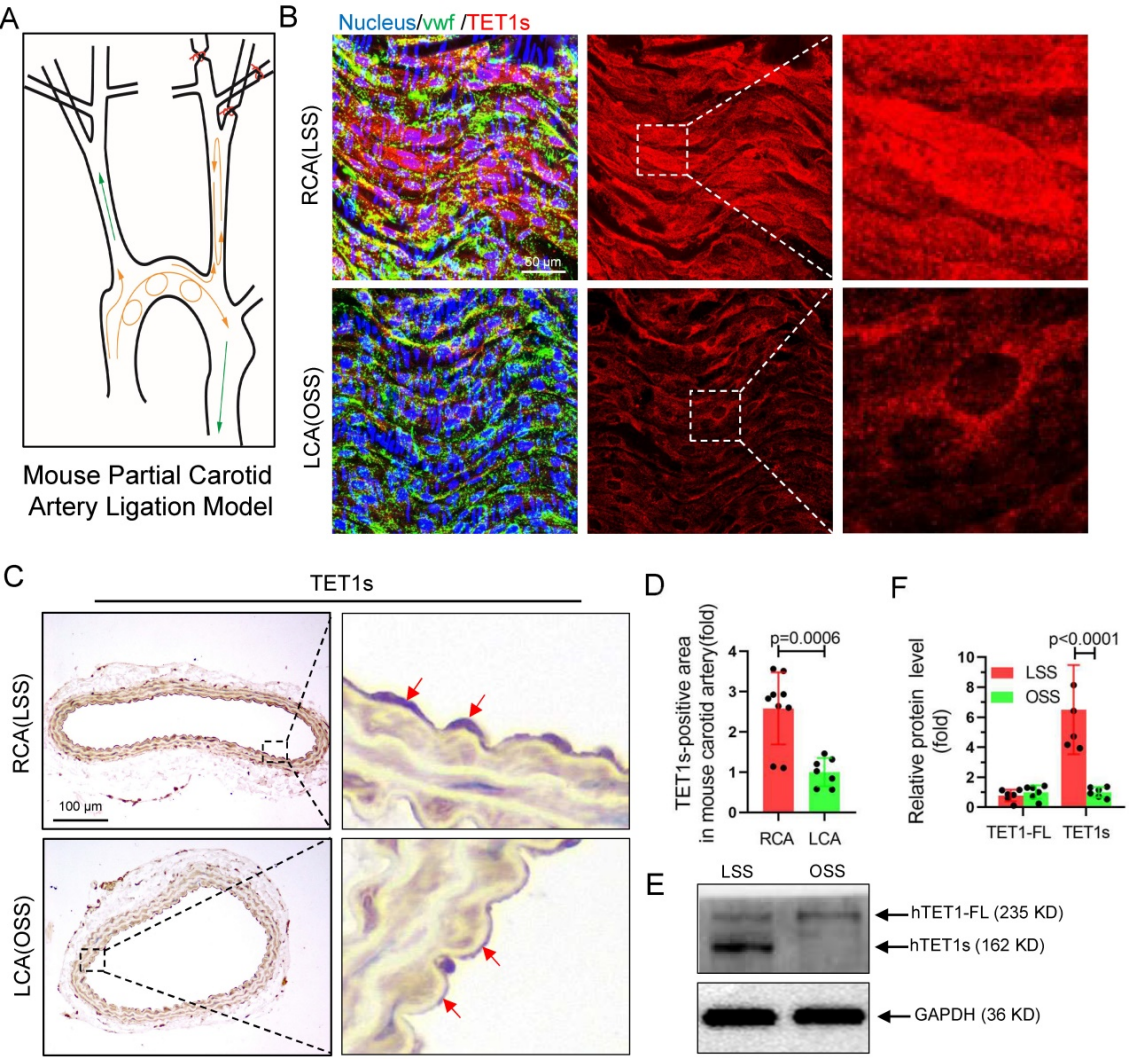
** TET1s expression** is **sensitive to flow shear stress.** (A) The mouse partial carotid artery ligation model. (B-D) *TET1^cs/cs^* mice LCA were ligated for 2 weeks. (B) Immunofluorescence staining and *en face* for TET1s in LCA and RCA ECs. (C-D) Immunohistochemical staining for TET1s in carotid artery slices and quantitative analysis of the TET1s-positive area; red arrows indicate the positive area in ECs (n>7 per group). (E-F) Implementation of OSS or LSS for p-HUVECs by parallel-plate flow chamber (PPFC) and quantitative analysis of TET1s and TET1-FL protein levels by WB (n>5 per group). (G-J) *ApoE^-/-^TET1^cs/cs^* mouse and *ApoE^-/-^ TET1^-/-^* mouse LCAs were ligated and fed a high-fat diet for 4 weeks. All data were presented as the mean ± SD.

To further investigate whether TET1s expression is sensitive to OSS in ECs, we constructed a partial carotid artery ligation model in *TET1^cs/cs^* mice as described in our previous report^22^. Ligation of the LCA created local oscillating blood flow, and no ligated RCA displayed unidirectional pulsatile flow (Figure 3A). The immunofluorescence (*en face*) (Figure 3B) and immunohistochemistry (Figure 3C) results showed that TET1s expression levels in ECs under OSS stimulation were significantly decreased in *TET1^cs/cs^* mice (Figure 3D, Figure S4A and Figure S5A) and the nuclear/cytoplasmic ratio (Figure S4B) of TET1s in ECs in ligated LCA was also significantly decreased compared with no ligated RCA. In addition, we also tested the expression levels and intracellular distribution of TET1 in ECs in the aortic arch (AA, exposed to OSS) and in the thoracic aorta (TA, exposed to LSS) by immunofluorescence (*en face*) and immunohistochemistry (Figure S5A, D). Similar results were found: OSS inhibited TET1s expression levels and decreased the nuclear/cytoplasmic ratio of TET1s in aortic ECs (Figure S5B-C, E). Furthermore, the immunofluorescence and WB results showed that OSS indeed inhibited TET1s expression levels in p-HUVECs with a parallel-plate flow chamber (PPFC) for 24 h (Figure 3E-F and Figure S6A-C).

**Figure 4:**
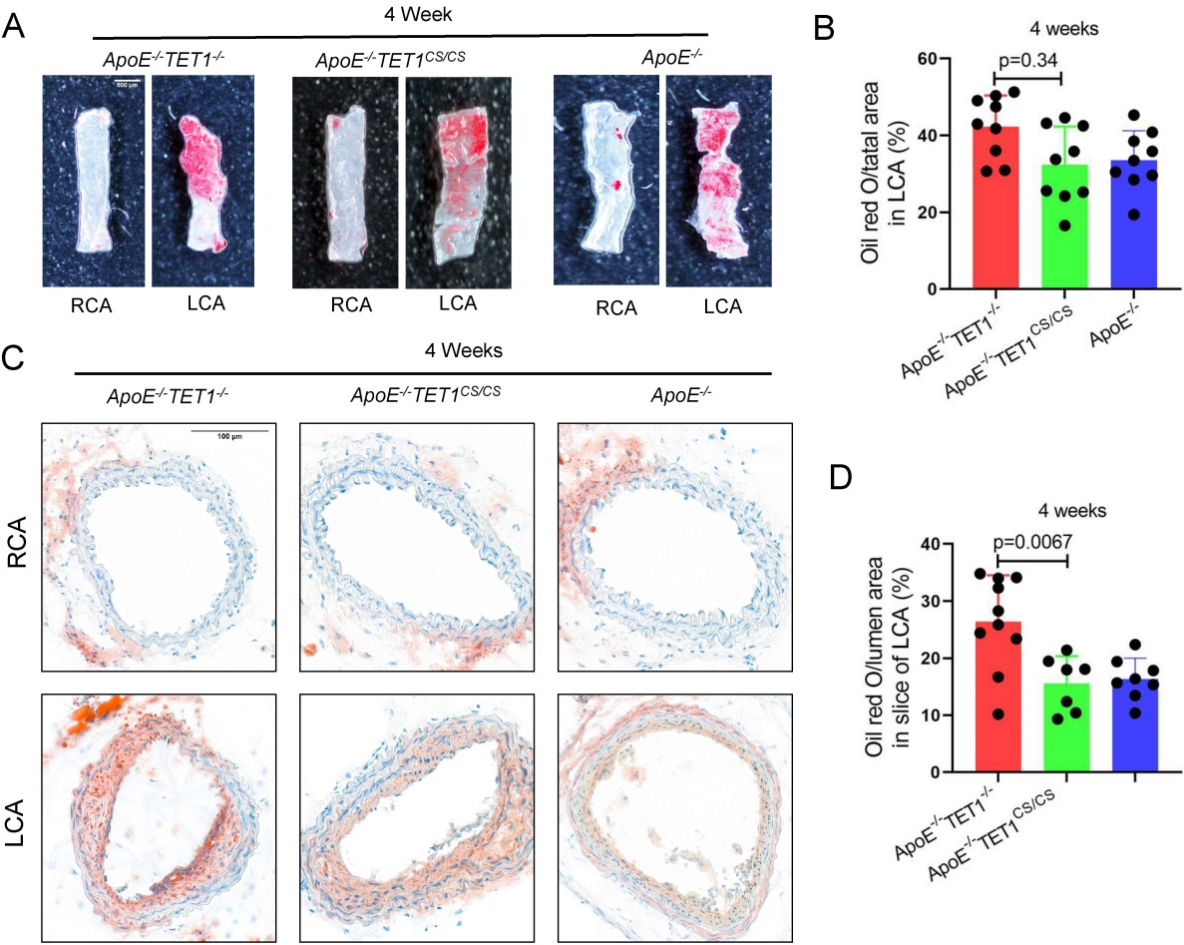
**TET1s mediates oscillatory shear flow-inducing atherosclerosis.** (A-D) *ApoE^-/-^TET1^cs/cs^* mouse and *ApoE^-/-^ TET1^-/-^* mouse LCAs were ligated and fed a high-fat diet for 4 weeks. (A-B) The lesion areas in the carotid artery were tested by ORO staining & *en face* and were analyzed (n>8 per group). (C-D) Oil red staining of the carotid artery and quantitative analysis of atherosclerotic plaque areas in the carotid artery (n>7 per group). All data were presented as the mean ± SD.

To ensure the function of TET1s in OSS-induced atherosclerosis, a partial carotid artery ligation model was used in *ApoE^-/-^TET1^cs/cs^*, *ApoE^-/-^TET1^-/-^* and *ApoE^-/-^* mice (Figure 3A) and then mice were fed a high-fat diet. We found that the size of plaques in *ApoE^-/-^TET1^-/-^* mice was significantly larger in the LCA examined *en face* (ORO staining) (Figure 4A-B) and LCA slices (ORO staining) (Figure 4C-D) relative to *ApoE^-/-^TET1^cs/cs^* and *ApoE^-/-^* mice fed a high-fat diet for 4 weeks. There was no difference for the high-fat diet at 1 week (Figure S8A-B).

These results show that OSS can inhibit TET1s expression levels in ECs and then induce atherosclerosis.

### TET1s enhances vascular endothelial barrier function *in vitro*

To identify the mechanism by which TET1s protect against disturbed flow-induced atherosclerosis, we performed RNA sequencing to test the global RNA levels of TET1s-overexpressing p-HUVECs and negative control p-HUVECs. Focal adhesion, cell-substrate junctions and cell-substrate adherens junction genes were in the top 20 pathways enriched by differential gene expression GO enrichment analysis with RNA-sequence data. KEGG enrichment analysis of differentially expressed genes revealed the top 20 enriched pathways, including leukocyte transendothelial migration, focal adhesion, fluid shear stress and atherosclerosis and adherens junctions (Figure S9A-B). These results imply that TET1s may regulate endothelial barrier function.

**Figure 5:**
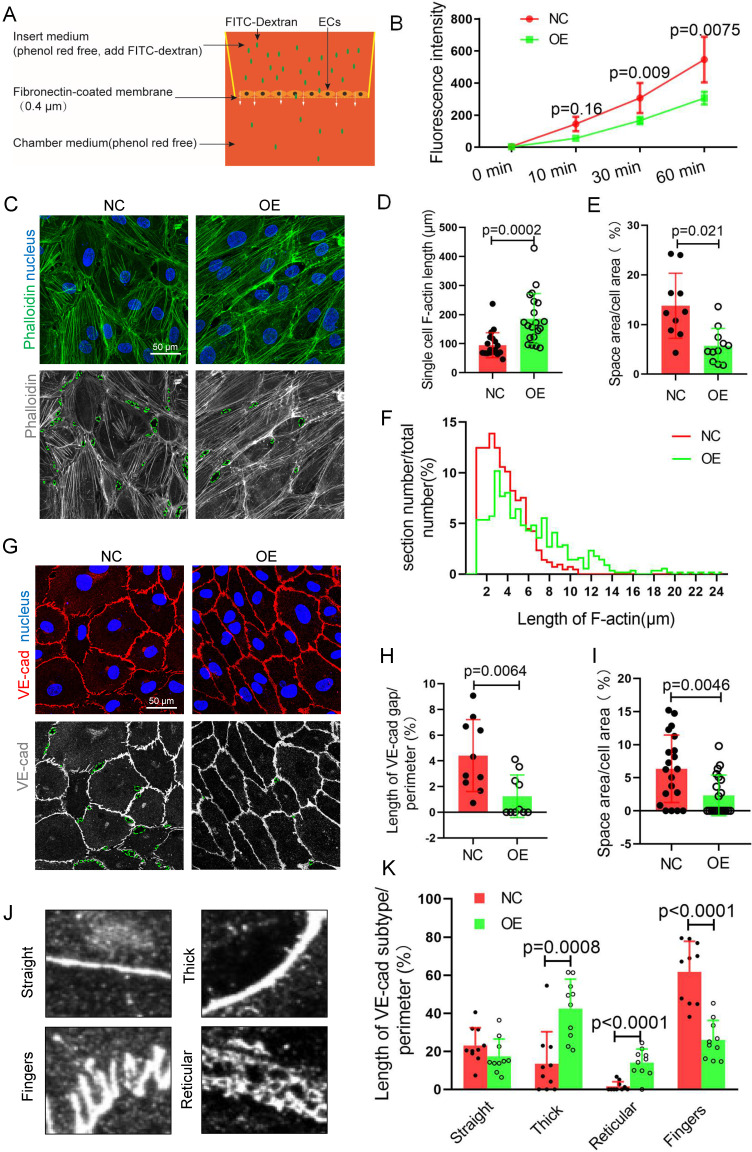
** TET1s enhances vascular endothelial barrier function.** (A) The model of Transwell assays for FITC-dextran trans-monolayer cells. (B-K) TET1s adenovirus-overexpressing and -negative adenovirus-transfected p-HUVECs and further experiments after 48 h. (B) The fluorescence intensity of the lower chamber medium was tested (n>6 per group). (C, G) Immunofluorescence staining for F-actin and VE-cadherin; the green dotted line indicates the intercellular space area. (D-F) Quantitative analysis of single-cell F-actin length, distribution of F-actin length and intercellular space area to image E (n>10 per group). (H-I, K) Quantitative analysis of VE-cadherin discontinuity, intercellular space area and ratio of VE-cadherin morphological categories to ImageJ (n>10 per group). (J) Several morphological categories of VE-cadherin. All data were presented as the mean ± SD.

To assess endothelial barrier function, FITC-dextran transmonolayer cells were tested and analyzed by Transwell assays with fluorescence intensity of the lower chamber medium (Figure 5A). The results showed that there was a decrease in the fluorescence intensity of the TET1s-overexpressing p-HUVEC group compared with the control group (Figure 5B), which indicated that TET1s can enhance vascular endothelial barrier function.

VE-cadherin, an adherens junction (AJ) molecule, is a key regulator of vascular permeability^30^,^31^. Cell-cell adhesion is linked through independent interactions with the actin cytoskeleton^32^,^33^. We speculated that TET1s enhances vascular endothelial barrier function relative to the cytoskeleton and VE-cadherin. For the cytoskeleton, we analyzed the ratio of the F-actin immunofluorescence area to the total cell area, single-cell F-actin length and the distribution of F-actin length (Figure 5C-D, F). To accurately describe the differences in VE-cadherin, as shown in Figure. 5J, several morphological categories were defined in previous studies^34^. Fingers and straight junctions represent unstable VE-cadherin. In contrast, thick reticular junctions represented stable VE-cadherin (Figure 5G). Thick and reticular junctions were the main VE-cadherin pattern (Figure 5K), and VE-cadherin discontinuity (Figure 5H) was decreased in p-HUVECs overexpressing TET1s, which indicated that TET1s increases the stability of VE-cadherin in p-HUVECs. Furthermore, TET1s also increased the p-HUVEC integrity (Figure 5E, I) by analyzing the monolayer p-HUVEC space area, as shown in Figure 5C and Figure 5G.

Therefore, TET1s enhances vascular endothelial barrier function by regulating the cell cytoskeleton and adhesion proteins *in vitro*.

### TET1s enhances the vascular intima barrier *in vivo*

**Figure 6:**
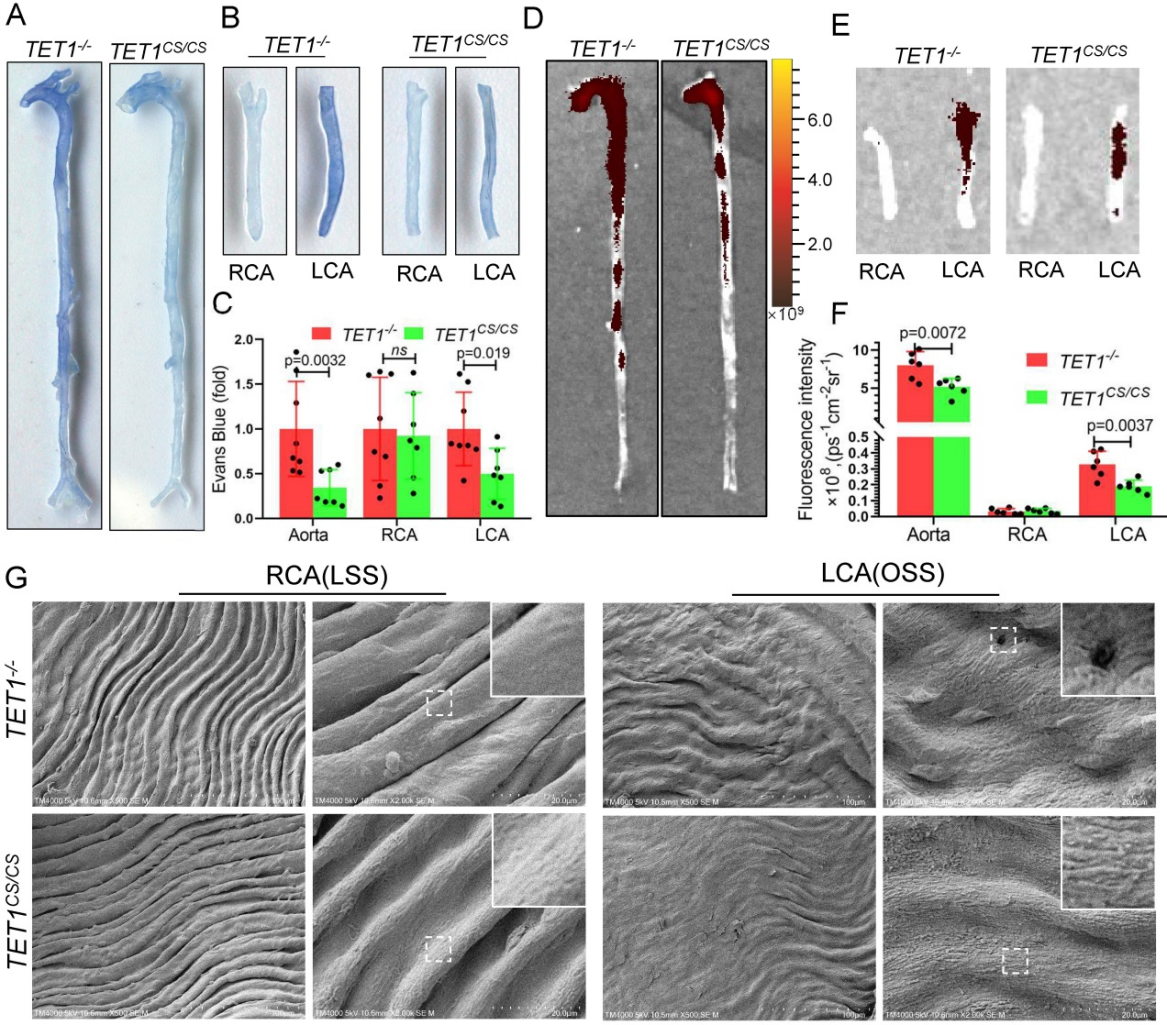
** TET1s enhances vascular intima barrier *in vivo*.** (A-G) *TET1^cs/cs^* and *TET1^-/-^*mouse LCAs were ligated with a regular diet for 2 weeks and subjected to further experiments. (A, B) Representative images showing Evans blue dye in aortas and carotid arteries. (C) The analysis of Evans blue dye for images A and B (n>6 per group). (D, E) Representative images showing nanoscale red blood cell membrane deposition in aortas and carotid arteries. (F) Analysis of fluorescence intensity for images D and E (n>6 per group). (G) The morphology of ECs in LCA and RCA by scanning electron microscopy (SEM). All data were presented as the mean ± SD.

To further study the role of TET1s in vascular endothelial barrier function *in vivo*, we assessed the permeability of ECs in the whole aorta and carotid artery of *TET1^cs/cs^* mice and *TET1^-/-^* mice with partial carotid artery ligation. Loss of TET1s increased Evans blue deposition in the aorta, particularly in the arterial root, aortic arch, abdominal aorta and ligation LCA, which were exposed in OSS (Figure 6A-C). Furthermore, nanoscale fluorescently labeled red blood cell membranes, described previously^35^, were also used to assess the permeability of the intima by tail vein injection, which showed similar results (Figure 6D-F).

Scanning electron microscopy (SEM) was used to directly observe the morphology of ECs in the aortic arch, thoracic aorta and carotid artery. The intima of the thoracic aorta and RCA exposed to LSS, presented a smooth surface. In contrast, the intima of the aortic arch and LCA, exposed to OSS, presented a rough surface with holes (signs of detachment). Compared with *TET1^-/-^* mice, the intima of the aortic arch and LCA of *TET1^cs/cs^* mice showed fewer holes (signs of detachment) between cells (Figure 6G and Figure S10A).

Collectively, these observations suggest that TET1s protects vascular intima barrier function from OSS by maintaining EC integrity.

### TET1s decreases red blood cell and inflammatory cell infiltration

**Figure 7:**
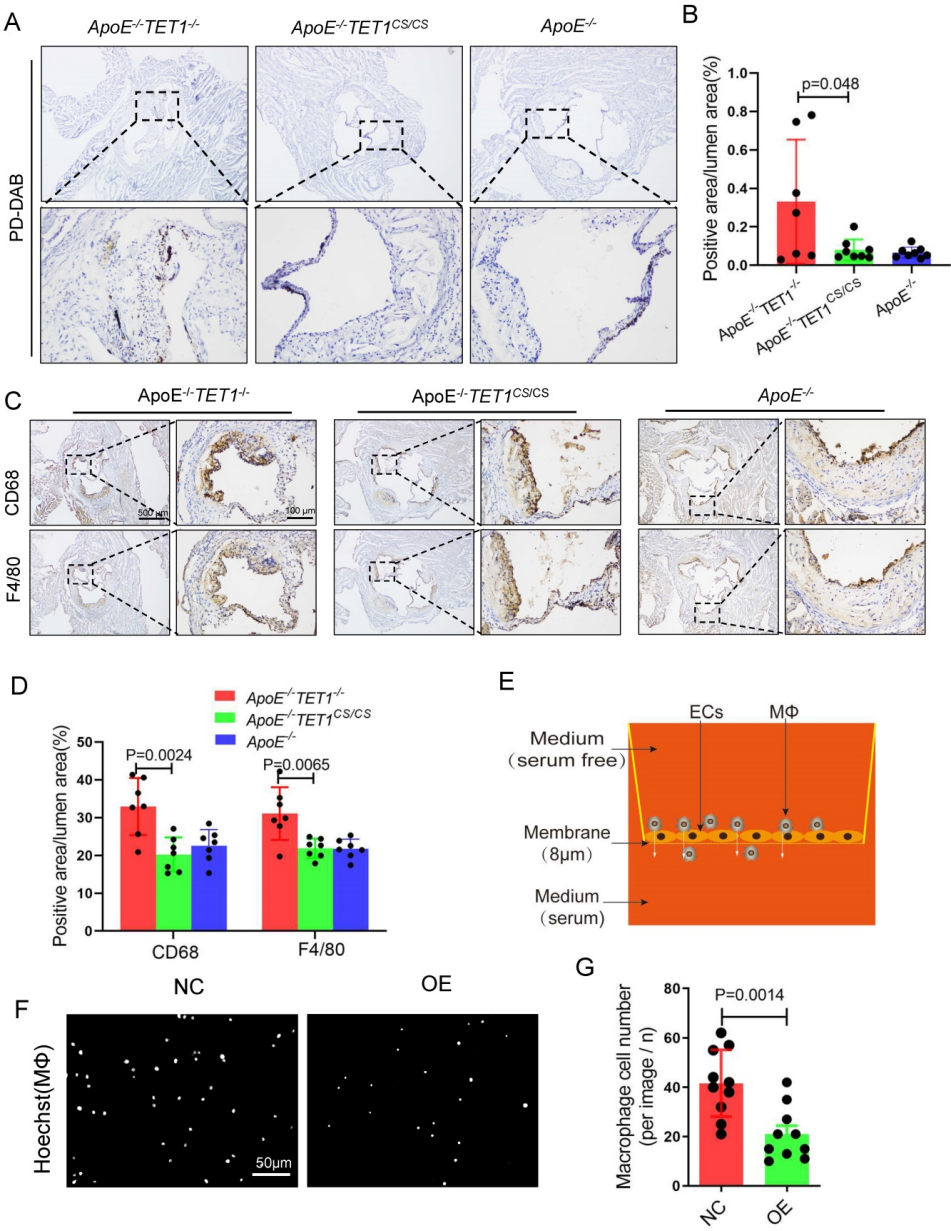
** TET1s decreases inflammatory cell infiltration.** (A-D) *ApoE^-/-^TET1^cs/cs^*, *ApoE^-/-^ TET1^-/-^* and *ApoE^-/-^* mouse LCAs were fed a high-fat diet for 4 weeks. The aortic roots were harvested and subjected to further experiments. (A) Representative PD-DAB staining for erythrocytes in aortic roots. (B) The positive area of erythrocyte infiltration was quantified by ImageJ software and calculated as the percentage of lumen (n>6 per group). (D) Representative immunohistochemical staining for macrophage-specific antigens CD68 and F4/80 in aortic roots. (E) The model of Transwell assays for macrophage cells through monolayer ECs* in vitro*. (F) Representative images showing macrophage cells through ECs monolayers from the membrane upper surface to the lower surface. (G) Analysis of macrophage number for image F (n=8 per group). All data are presented as the mean ± SD.

Red blood cell, leukocyte and lipid transendothelial migration into the subendothelial layer can play a critical role in the initial stage of atherosclerosis development, which is associated with the permeability of the EC layer^6^,^36^. We used Perl's + diaminobenzidine (DAB) staining to specifically detect iron derived from red blood cells as previously described^6^. Erythrocyte outflow in the plaques of* ApoE^-/-^TET1^-/-^* mice was significantly increased compared with that of *ApoE^-/-^TET1^cs/cs^* mice (Figure 7A-B). Inflammatory cells containing macrophages and neutrophils in the arterial root of *ApoE^-/-^TET1^cs/cs^* mice and *ApoE^-/-^TET1^-/-^*mice fed a high-fat diet for 4 weeks were evaluated by immunohistochemical analysis. The results revealed that the expression of CD68 and F4/80 (Figure 7C-D), markers of macrophages, and CD11b and Ly6G (Figure S11A-B), markers of neutrophils, in the plaques of *ApoE^-/-^TET1^cs/cs^* mice were significantly decreased compared with those of *ApoE^-/-^TET1^-/-^* mice. These data show that TET1s deletion impairs the vascular intima barrier.

We further used Transwell assays (Figure 7E) to test the obstructive ability of TET1s-overexpressing p-HUVECs and negative control p-HUVECs. The results showed that TET1s-overexpressing p-HUVECs significantly decreased the transendothelial migration of macrophage cells compared with negative control p-HUVECs (Figure 7F-G).

Therefore, TET1s protects against disturbed flow-induced atherosclerosis by enhancing vascular endothelial barrier function and decreasing inflammatory cell infiltration.

### Connexin 40 mediates TET1s-induced endothelial barrier reinforcement

**Figure 8:**
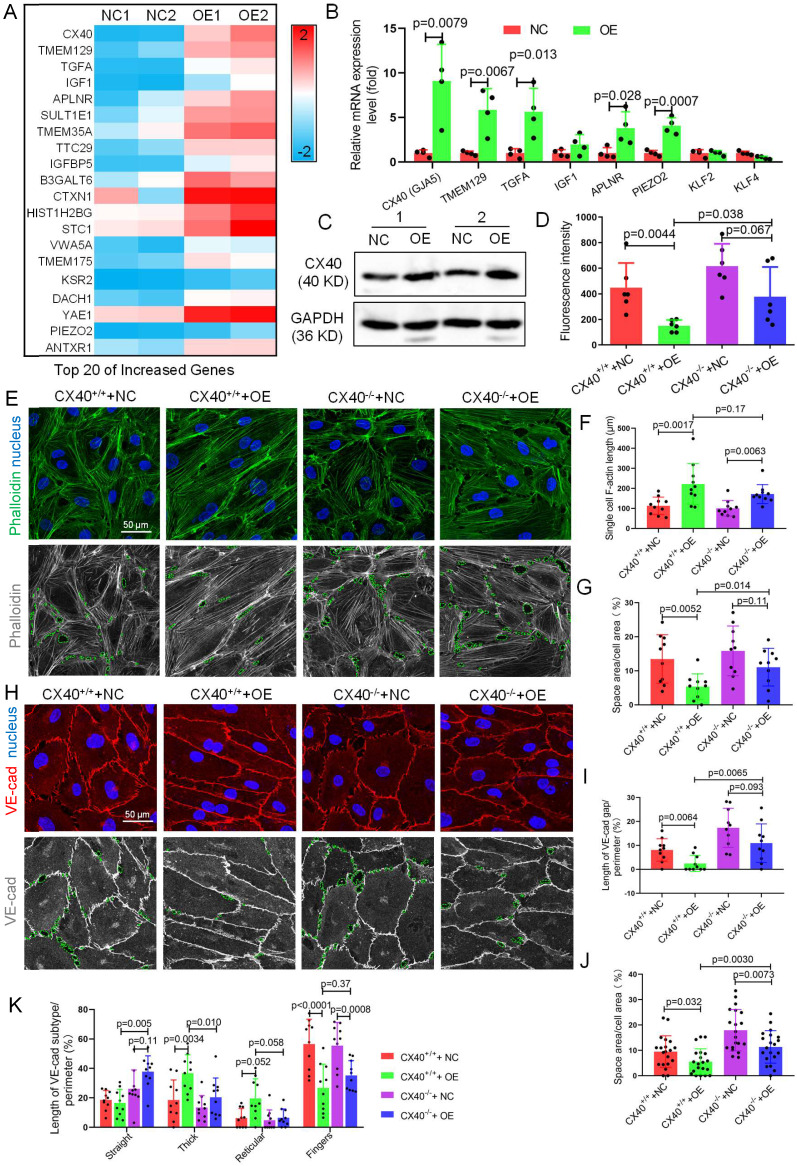
** CX40 mediates TET1s-induced endothelial barrier reinforcement.** (A) Heatmap of the top 20 selected upregulated genes by RNA sequencing. (B) RT-qPCR was used to test the mRNA levels of the top 5 upregulated genes from RNA-seq and three hemodynamic-sensitive genes. (C) The CX40 protein expression level was quantified by WB (n=6 per group). (D-L) Stable CX40^-/-^ p-HUVECs were generated by transfecting human connexin 40-specific CRISPR/Cas9 KO plasmids. Then, TET1s-adenovirus was used to transfect CX40^-/-^ and CX40^+/+^ p-HUVECs to generate CX40^+/+^+NC, CX40^+/+^+OE, CX40^-/-^+NC and CX40^-/-^+OE p-HUVECs. (D) The fluorescence intensity of the lower chamber medium was tested as described in Fig. 3C (n>6 per group). (E, H) Immunofluorescence staining for F-actin and VE-cadherin. The green dotted line indicates the intercellular space area. (F-G) Quantitative analysis of single-cell F-actin length and intercellular space area to image E (n>10 per group). (I-K) Quantitative analysis of VE-cadherin discontinuity, intercellular space area and ratio of VE-cadherin in several morphological categories to image H (n>10 per group). All data were presented as the mean ± SD.

To identify the mechanism by which TET1s enhance endothelial barrier function, we further investigated the gene expression changes revealed by the RNA-sequence data and performed heatmap analysis. we found the CX40 expression increase the most in all increased gene (Figure 8A). As a key component of gap junction, CX40 contributes to maintain endothelial barrier and permeability^37^,^38^. Hence, we speculated that CX40 may be the major target of TET1s-induced endothelial barrier reinforcement.

The mRNA levels of CX40 (gap junction protein) in TET1s-overexpressing p-HUVECs increased 11-fold compared with the negative control. Then, we verified the data by RT-qPCR and WB and found that TET1s overexpression significantly increased CX40 expression (Figure 8B-C). Besides, we also assessed the effect of TET1s deficiency on CX40 expression *in vivo*. We found the loss of TET1s decrease the CX40 expression in *TET1^-/-^* mice relative to *TET1^cs/cs^* and *WT* mice in oscillatory and laminar shear flow region of aorta endothelial layer and with partial carotid artery ligation (Figure S12).

To identify CX40 in TET1s-induced endothelial barrier reinforcement, we first used human CX40-specific CRISPR/Cas9 KO plasmids to generate stable CX40^-/-^ and CX40^+/+^ p-HUVECs. Then, we used TET1s adenovirus to transfect CX40^-/-^ and CX40^+/+^ p-HUVECs to generate CX40^+/+^+NC, CX40^+/+^+OE, CX40^-/-^+NC and CX40^-/-^+OE p-HUVECs, respectively. The results showed that the permeation of FITC-dextran in the CX40^-/-^+OE p-HUVEC group was significantly increased compared with that in the CX40^+/+^+OE group (Figure 8D).

Single-cell F-actin length was not different between the CX40^+/+^+OE and CX40^-/-^+OE p-HUVEC groups (Figure 8F). However, the intercellular space area (Figure 8G) in the CX40^-/-^+OE group was significantly increased compared with that in the CX40^+/+^+OE p-HUVEC group. For VE-cadherin, the VE-cadherin discontinuity and intercellular space area in the CX40^-/-^+OE group were significantly increased compared with those in the CX40^+/+^+OE p-HUVEC groups (Figure 8H-J). In several morphological categories of VE-cadherin, thick and reticular junctions in the CX40^-/-^+OE group were significantly decreased compared with those in the CX40^+/+^+OE p-HUVEC groups (Figure 8K). Straight junctions were significantly increased, and finger junctions were not different (Figure 8K).

Taken together, these data demonstrate that the deletion of CX40 partly relieves TET1s-induced endothelial barrier reinforcement in p-HUVECs.

### TET1s overexpression increases CX40 expression by inhibiting histone deacetylation

**Figure 9:**
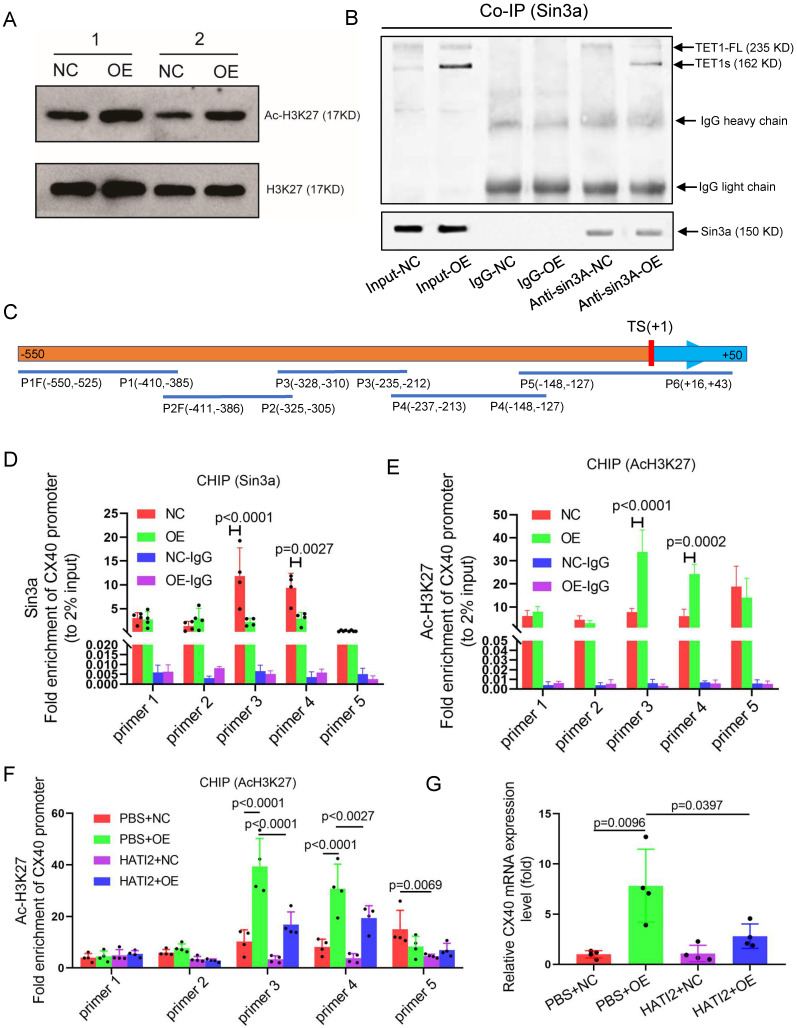
** TET1s increases CX40 expression by inhibiting histone deacetylation on the promoter of CX40.** (A-B, D-E) p-HUVECs were transfected with TET1s-overexpressing adenovirus and negative control adenovirus and further tested after 48 h. (A) The global protein levels of ac-H3K27 and H3K27 in p-HUVECs were tested by Western blot (n=6 per group). (B) Sin3a interaction with TET1s and TET1-FL was analyzed by Co-IP (n=3 per group). (C) Schematic of human CX40 promoter and CHIP-qPCR products. TS indicates transcriptional start; P1-P5 indicates primer 1-primer 5; F indicates forward primer, R indicates reversed primer. (D-E) ChIP-qPCR was used to test Sin3a and ac-H3K27 enrichment in the CX40 promoter (-550 bp to +43 bp) (n=4 per group). (F-G) p-HUVECs were transfected with TET1s-overexpressing adenovirus and negative control adenovirus for 48 h and added HATI2 to media. (F) ChIP-qPCR was used to test ac-H3K27 enrichment in the CX40 promoter. (G) The CX40 mRNA levels were tested by RT-qPCR (n=4 per group). All data were shown as the mean ± SD.

Previous studies have already suggested that TET1 promotes gene expression in ESCs by binding TSSs in promoters and oxidizing methylated cytosine into 5-hydroxymethylcytosine (5hmC)^39^. The isoforms of TET1 and TET1s retain the catalytic domain but lack the DNA-binding CXXC domain^18^, so whether TET1s has an oxidation function remains unclear. We found that TET1s overexpression in p-HUVECs did not cause a global difference in 5hmC and 5mC levels by dot blot assay (Figure 9A-B and Figure S13A-B). To explore the mechanism of the increase in CX40 expression, bioinformatic analysis showed that the CX40 promoter lacked an obvious CpG island and revealed only 6 disconnected CpG sites (Figure 9C). We also found that the methylation status of 6 CpG sites in the CX40 promoter was not changed in the TET1s overexpression group by pyrosequencing assays (Figure 9D). Thus, it does not underlie the oxidation activity of TET1s in inducing CX40 expression.

Recent studies have demonstrated that TET1 can participate in histone deacetylation to modulate transcriptional repression via interaction with the scaffold protein Sin3a^14^. We tested global acetylated histones by Western blotting and chose H3K27 acetylation as a typical acetylated histone. Surprisingly, TET1s overexpression in p-HUVECs significantly increased H3K27 acetylation levels but did not decrease H3K27 acetylation levels (Figure 9E). Next, we found that TET1s can also interact with Sin3a, which share the characteristic of TET1-FL (Figure 9F). Given that TET1s is the absolute predominant transcript in ECs compared with TET1-FL, TET1s is more likely to bind Sin3a directly. A previous study showed that TET1 recruitment of Sin3a to DNA binding sites is necessary for the deacetylation function of the Sin3a/HDAC complex^40^; however, TET1s lacks the CXXC domain, a DNA-binding module^18^. Based on the above results, we speculated that because TET1s lacks the CXXC domain, TET1s interaction with Sin3a not only fails to recruit Sin3a to the DNA binding sites but also competitively inhibits the binding of TET1-FL, leading to an increase in acetylated histones in the CX40 promoter, which further promotes CX40 expression upregulation. To confirm our hypotheses, we performed ChIP-PCR to analyze Sin3a colocalization with the CX40 promoter and the H3K27 acetylation levels of the CX40 promoter. The PCR product covered the CX40 promoter from -550 bp to +43 bp (Figure S14A). The ChIP-PCR results showed that Sin3a colocalization with the CX40 promoter was decreased in the TET1s overexpression groups (Figure 9G). The H3K27 acetylation levels of the CX40 promoter in TET1s-overexpressing p-HUVECs were significantly increased compared with those in negative control p-HUVECs (Figure 9H).

To confirm that the histone acetylation level in the CX40 promoter region is a key factor in TET1s-induced CX40 expression, we inhibited the activity of histone acetyltransferase using histone acetyltransferase inhibitor II (HATI2) in p-HUVECs. The histone acetylation level in the CX40 promoter region was significantly decreased in the HATI2 group compared with the PBS group after 48 h, as determined by ChIP-PCR (Figure S15A). HATI2 implementation significantly remitted TET1s-induced CX40 expression increase (Figure 9I). This showed that the histone acetylation level in the CX40 promoter region was indeed a key factor in TET1s-induced CX40 expression.

These results imply that TET1s increases CX40 expression, which depends upon the inhibition of histone deacetylation in the CX40 promoter by its interaction with Sin3a, but not TET1s oxidation function.

## Discussion

Atherosclerotic lesions tend to occur in the curvature and bifurcation of the vasculature with complex hemodynamic conditions^41^. Since ECs are routinely exposed to mechanical forces, they are highly responsive to various hemodynamic stimuli^4^. These stimuli may be some of the major reasons for atherosclerotic development in the initiation stage. The so-called mechanoadaptors conduct mechanical signals from a mechanosensor to a receptor molecule and unlock downstream progress^42^. In our study, we also found that plaques were more prevalent in OSS areas, including the aortic arch, abdominal aorta and ligated LCA (Figure 4A-D). Our results showed that TET1s is sensitive to hemodynamic stimuli in ECs (Figure 3B-E, Figure S4A-B, Figure S5 and Figure S6) and deletion of TET1s exacerbates disturbed flow-induced atherosclerosis (Figure 1B-G and Figure 4). Thus, TET1s may be a mechanoadaptor and conduct signals to endonuclear receptors and regulate downstream gene expression.

Functional and structural changes of endothelial barrier are some of the major early atherosclerotic features^43^. ECs, as a unique barrier separating the vascular wall from the blood, control the exchange of substances between the vessel lumen and the vascular wall in part through the dynamic regulation of endothelial cell-cell junctions, which play an important role in maintaining vascular homeostasis. The shear stress of blood flowing on the surfaces of endothelial cells regulates various transport pathways, including tight junctions, adherens junctions, vesicles and leaky junctions^44^. Increasing evidence has shown that hemodynamic stress can directly or indirectly regulate the vascular endothelial barrier. Transendothelial leakage of macromolecules, including LDL, is most frequently located around vascular branch points^45^. In contrast, nonbranch areas show a decrease in the leakage of macromolecules. The permeability of branch sites is approximately four times higher than that of the nonbranch areas^46^. According to a recent study, flow perturbation induces breaches of the arterial intima, which drive the recruitment of leucocytes at sites of disturbed arterial flow^6^. In this study, we illustrate that these sites in the aortic arch, abdominal aorta and ligated LCA, which were exposed to OSS, have a significantly increased permeability of the vascular endothelium compared with the thoracic aorta and nonligated RCA (Figure 6A-F). In addition, we also found that hemodynamic stress influences the surface morphology of ECs (Figure 6G and Figure S10). These results further showed that OSS significantly increased the permeability of the vascular endothelium and that LSS had the opposite effect.

VE-cadherin can also be directly activated by a twisting magnetic force^47^. Similarly, VE-cadherin also undergoes the same tension change when exposed to different hemodynamic shear stresses. Studies show that cell junction-related proteins, including VE-cadherin, F-actin and occludin^48^,^49^, could be mechanosensing molecules, mechanoadaptors or downstream receptor molecules. Our results showed that upregulation of TET1s expression enhances vascular endothelial barrier function *in vitro* (Figure 5B-K). Deletion of TET1s increased inflammatory cell and red blood cell infiltration *in vivo* (Figure 7A-D, and Figure S11). In this process, TET1s, as a mechanoadaptor, mediates hemodynamic shear stress regulating the vascular endothelial barrier.

Vascular permeability can be regulated by local and acute hemodynamic forces through remodeling of endothelial cell-cell junctions and the cytoskeleton^50^. Studies have shown that hemodynamic shear stress-responsive signaling pathways mediate endothelial cytoskeletal and intercellular junction remodeling^51^. In this study, to uncover the role of TET1s in the vascular endothelial barrier, endothelial cytoskeletal, F-actin, and intercellular junction, VE-cadherin, were investigated to assess changes in the vascular endothelial barrier (Figure 5C, G and Figure 8E, H,). We analyzed the properties of F-actin and VE-cadherin after overexpressing TET1s in ECs and verified that TET1s enhances vascular endothelial barrier function (Figure 5D-F, J-H).

As a truncated TET1, TET1s still shares catalytic activity with TET1-FL to oxidize 5mC to 5hmC^18^. However, TET1-FL also displays a distinct and independent demethylation function in the regulation of the luteinizing hormone gene (Lhb), which may involve the recruitment of histone H3K27 methyltransferases^52^. In fact, such effects have been reported in ESCs, and TET1-FL appears to recruit histone-modifying enzymes via its interaction with the Sin3A complex^14^. Here, we demonstrate that TET1s overexpression does not cause a global increase in 5hmC levels (Figure S13C-D) or a local 5hmC level increase in the CX40 promotor (Figure S13E-F). We found that both TET1s and TET1-FL can interact with Sin3A (Figure 9B). Full-length TET1 interacts with the Sin3a complex and further represses gene transcription via histone deacetylation of histone deacetylase (HDAC) in the complex^53^. However, TET1s upregulation significantly increases global acetylation H3K27 levels (Figure 9A) and causes an increase in local acetylation H3K27 levels and a decrease in local Sin3a levels in the CX40 promotor (Figure 9E). Thus, it seems that TET1s has the opposite function to that of TET1-FL, that is, TET1s promotes CX40 expression by increasing H3K27acetylation levels in the CX40 promotor. In fact, we also verified that TET1s upregulation increased CX40 expression (Figure 8A-C, Figure S12). The most likely explanation is that the lack of a CXXC domain in this short isoform leads to a failure of the Sin3a complex to locate a suitable position in the promotor, which block deacetylated function of Sin3a complex, and maintain high acetylated histone level in CX40 promotor.

The reorganization of endothelial cell-cell junctions, specifically the tight and adherens junctions, and cytoskeletal modifications control the endothelial barrier^54^,^55^. Emerging evidence does suggest that gap junction also contribute to maintain endothelial barrier and permeability. Endothelial Cx40 regulate neutrophil infiltration in cardiac reperfusion^37^ and monocyte recruitment in the context of atherosclerosis^38^. Cx40 also contributes to lung endothelial permeability by regulation of Rho-associated protein kinase 1 (ROCK1) and myosin light chain 20 (MLC20)^56^. In this study, we showed CX40 is require for overexpressing TET1s-induced endothelial barrier enhancement (Figure 8D). The mechanism may be associated with VE-cadherin and F-actin (Figure 8E-K). It has been reported, as intercellular ion channels, CX40 regulates calcium uptake in coronary endothelial cells^57^ and calcium concentration is required for cytoskeletal modifications and reorganization of endothelial cell-cell junctions. Thus, it is possible that CX40 contribute to vascular endothelial barrier by calcium concentration to regulate cytoskeletal modifications and reorganization of endothelial cell-cell junctions.

In this study, we show that global deletion of TET1s accelerates atherosclerotic development by impairing endothelial barrier function in response to OSS (Figure 1C-G and Figure 2). Unfortunately, we did not apply a specific endothelial knockout model in mice. TET1s is not only expressed in ECs, but also expressed in SMCs and macrophages. Given the atherosclerotic development being also closely linked SMCs and macrophages, the acceleration effect of TET1s on atherosclerotic development can be not whole attributed to endothelial TET1s. We have proved that TET1s has less expression in smooth muscle cell and macrophages than ECs (especially being exposed on LSS) (Figure S7). *In vitro*, overexpressing TET1s in ECs enhance the vascular endothelial barrier function (Figure 5B-K). Deletion of TET1s increase the vascular intimal permeability *in vivo* (Figure 6A-F, Figure 7A-D and Figure S10A-B). And endothelial barrier injuring is the major reason of early atherosclerotic development^43^. Thus, endothelial TET1s is, at least, an important factor in flow shear stress-regulated atherosclerosis development.

Due to TET1s gene shares the vast majority of exon with TET1-FL, it is difficult for us to solely knockout TET1s gene. Both TET1^-/-^mice, lack of TET1-FL and TET1s, and TET1cs/cs mice, lack of TET-FL were introduced to research the function of TET1s. To investigate the role of TET1s in the development of atherosclerosis, two double knockout mice, ApoE^-/-^TET1^-/-^ and ApoE^-/-^TET1^cs/cs^ were constructed by TET1^-/-^mice and TET1cs/cs mice crossing with ApoE^-/-^ mice, respectively. Two kinds of mice are both lack TET1-FL, and their difference is only the TET1s expression. For the same reason, TET1s specific antibody is also difficult to acquire. So we choose TET1 antibody as second best. In TET1s immunostaining assay, TET1^cs/cs^ mice is applied to avoid cross reaction of TET1 antibody.

In conclusion, this study demonstrates that TET1s is sensitive to hemodynamic stimuli in ECs and OSS inhibits TET1s expression. TET1s retards oscillatory shear flow-inducing atherosclerosis. Regarding the mechanism for this effect, we find that TET1s enhances the endothelial cell barrier and inhibits inflammatory and red blood cell infiltration into the subendothelial layer by increasing CX40 expression through increases histone acetylation levels in the CX40 promoter via its interaction with Sin3a.

## Supplementary Material

Supplementary figures and tables.

## Abbreviations

5hmC: 5-hydroxymethylcytosine
5mC: 5-methylcytosine
AA: aortic arch
AJ: adherens junction
BCA: bicinchoninic acid
BSA: bull serum albumin
Cas9: CRISPR associated protein 9
CHIP: chromatin immunoprecipitation
Co-IP: co- immunoprecipitation
CRISPR: clustered regularly interspaced short palindromic repeats
CXXC: Cys-Xaa-Xaa-Cys
CX40: connexin 40
DAPI: 4',6-diamidino-2-phenylindole
DNA: deoxyribonucleic acid
ECM: EC medium
ECs: endothelial cells
EGS: endothelial growth supplement
ESCs: embryonic stem cells
FCS: fetal calf serum
FITC: fluorescein isothiocyanate
GAPDH: glyceraldehyde-phosphate dehydrogenase
GO: Gene Ontology
HATI2: histone acetyltransferase inhibitor II
HDR: homology-directed DNA repair
IF: Immunofluorescence
HDAC: histone deacetylase
IP: immunoprecipitation
KEGG: Kyoto Encyclopedia of Genes and Genomes
LCA: left carotid artery
LSS: laminar shear stress
NC: negative control
OE: overexpression
ORO: Oil Red O
OSS: oscillatory shear stress
p-HUVECs: primary human umbilical vein endothelial cells
qPCR: quantitative polymerase chain reaction
RCA: right carotid artery
RNA: ribonucleic Acid
qRT-PCR: real-time quantitative reverse transcription PCR
SD: standard deviation
SDS: sodium dodecyl sulfate
SDS-PAGE: sodium dodecyl sulfate polyacrylamide gel electrophoresis
SMCs: smooth muscle cells
SEM: scanning electron microscopy
TA: thoracic aorta
TET1: Tet methylcytosine dioxygenase 1
TET1s: short isoform of TET1
TET1-FL: full-length TET1
TET2: Tet methylcytosine dioxygenase 2
TET3: Tet methylcytosine dioxygenase 3
TS: transcriptional start
WB: western blot
