# Supplementary material for: TET1s deficiency exacerbates oscillatory shear flow-induced atherosclerosis
Source: Int J Biol Sci. 2022 Feb 28;18(5):2163–80. doi: 10.7150/ijbs.69281 (PMC8935216; doi:10.7150/ijbs.69281)
Supplement: Supplementary file 1 — Supplementary figures and tables. [file ijbsv18p2163s1.pdf]

Supplementary files

TET1s deficiency exacerbates oscillatory shear flow-induced atherosclerosis

Kai Qu[1], Caihong Wang[1], Lu Huang[1], Xian Qin[1], Kun Zhang[1], Yuan Zhong[1], Qingfeng Ma[1], Wenhua Yan[1], Tianhan Li[1], Qin Peng[2], Yi Wang[1], Hans Gregersen[1,3], Chaojun Tang[4], Juhui Qiu[1,*], Guixue Wang[1,*]

1. Key Laboratory for Biorheological Science and Technology of Ministry of Education, State and Local Joint Engineering Laboratory for Vascular Implants, College of Bioengineering Chongqing University, Chongqing, China;
2. Institute of Systems and Physical Biology, Shenzhen Bay Laboratory, Shenzhen, China;
3. GIOME, Department of Surgery, the Chinese University of Hong Kong, Hong Kong, China.
4. Cyrus Tang Hematology Center, Collaborative Innovation Center of Hematology, Soochow University, Suzhou, China.

*Corresponding author. Email: wanggx@cqu.edu.cn (Gui-xue Wang); jhqiu@cqu.edu.cn (Ju-hui Qiu)

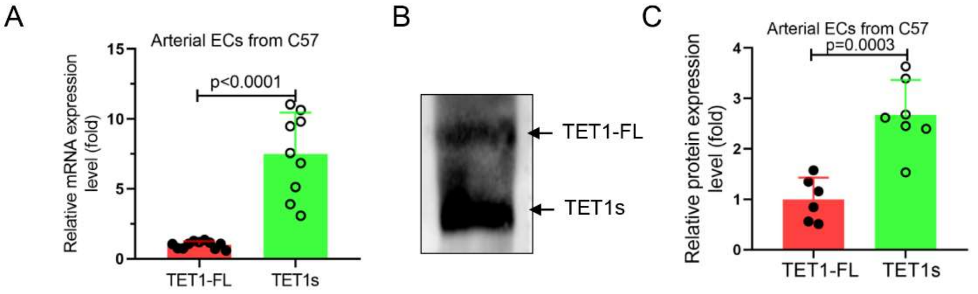

Figure S1. TET1s is the predominant transcript compared to TET1-FL in arterial ECs from C57 mice. (A-C) All ECs separated from the aorta of C57mice. (A) RT-qPCR was used to test the mRNA levels of TET1s and TET1-FL(n>6 per group). (B-C) The TET1s and TET1-FL protein expression level was quantified by WB (n>6 per group). All data were presented as the mean ± SD.

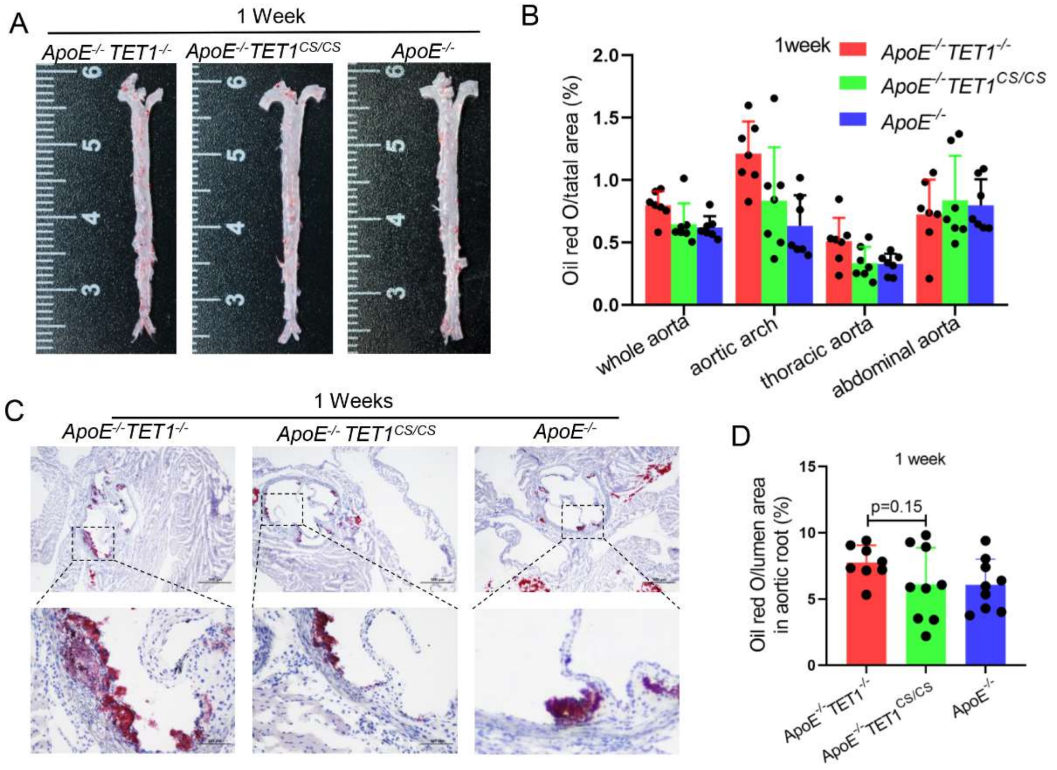

Figure S2. The plaques in ApoE$^{-/-}$TET1cs/cs mice was no difference compared with ApoE$^{-/-}$ TET1$^{-/-}$mice fed a high-fat diet for 1 week. (A-D) ApoE$^{-/-}$ TET1$^{-/-}$, ApoE$^{-/-}$ TET1$^{cs/cs}$ and ApoE$^{-/-}$ mice (8 weeks old) were fed a high-fat diet for 1 weeks. (A) The aortic plaques of ApoE$^{-/-}$ TET1$^{-/-}$, ApoE$^{-/-}$ TET1$^{cs/cs}$ and ApoE$^{-/-}$ mice were tested by red oil staining and en face microscopy. (B) The lesion areas in the whole aorta, aortic arch, thoracic aorta, and abdominal aorta sections were

analyzed (n>7 per group). (C-D) Representative photomicrographs of aortic root slice red oil staining and quantitative analysis of atherosclerotic plaque areas in the aortic root (n>7 per group). All data were presented as the mean ± SD.

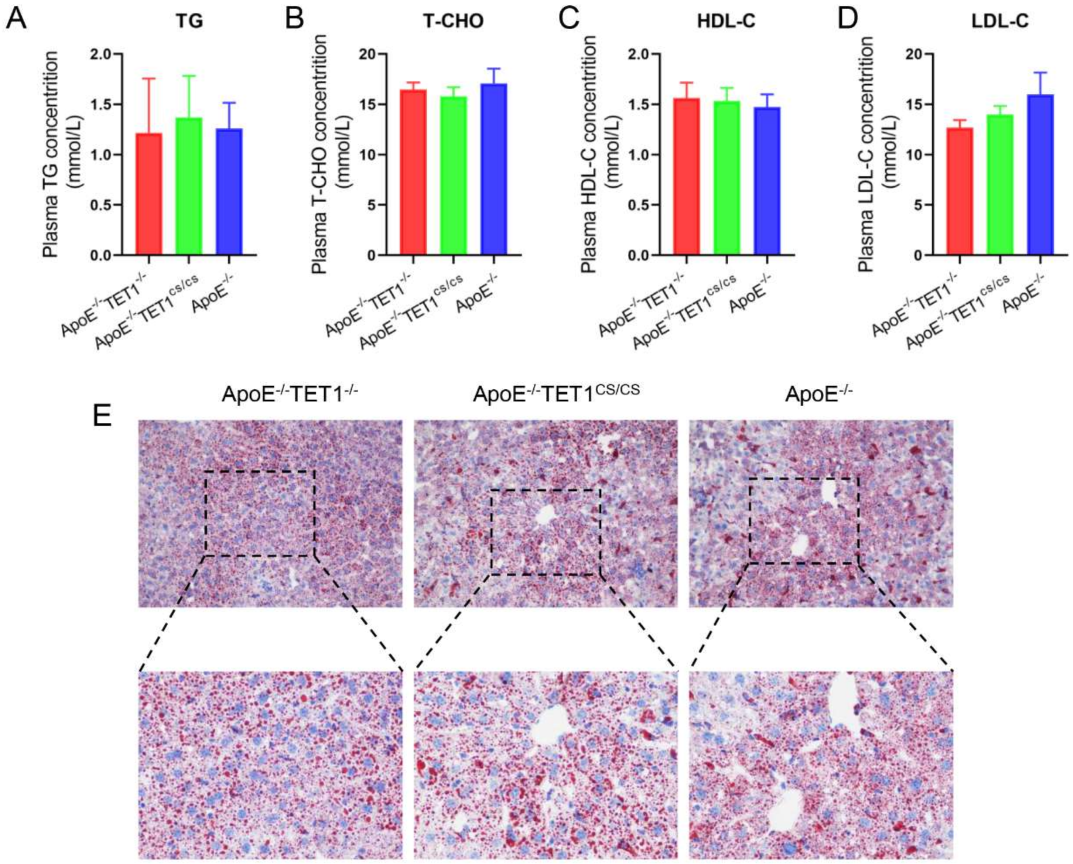

Figure S3. Deletion of TET1s has no significant change in lipid metabolism in ApoE$^{-/-}$ mice. (A-E) ApoE$^{-/-}$TET1$^{-/-}$, ApoE$^{-/-}$TET1$^{CS/CS}$ and ApoE$^{-/-}$ mice fed with high-fat diet for 12 weeks. (A) Quantitative analyze in triglyceride (TG) of ApoE$^{-/-}$TET1$^{-/-}$, ApoE$^{-/-}$TET1$^{CS/CS}$ and ApoE$^{-/-}$ mice fed with high-fat diet for 12 weeks. (A-D) Quantitative analyze triglyceride (TG), total cholesterol (T-CHO), LDL-C and HDL-C of ApoE$^{-/-}$TET1$^{-/-}$, ApoE$^{-/-}$TET1$^{CS/CS}$ and ApoE$^{-/-}$ mice (E) The representative images with hepatic slice staining with oil red O of ApoE$^{-/-}$TET1$^{-/-}$, ApoE$^{-/-}$TET1$^{CS/CS}$ and ApoE$^{-/-}$ mice. All data are presented with mean value and standard deviation (SD).

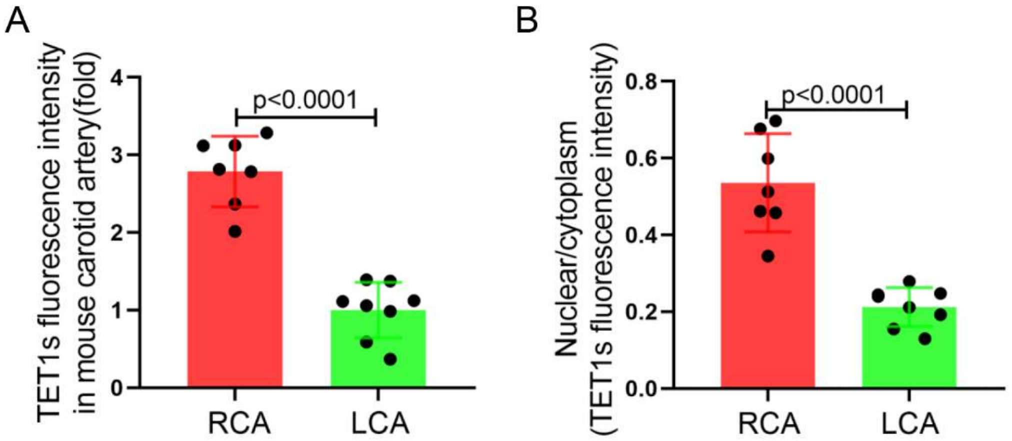

Figure S4. OSS stimulation was decreased the expression and the nuclear/cytoplasmic ratio of TET1s in carotid ECs in TET1$^{cs/cs}$ mice. (A-B) TET1$^{cs/cs}$ mice LCA were ligated for 2 weeks. (A) Quantitative analysis of TET1s fluorescence intensity in LCA and RCA ECs to fig.3B. (B) Quantitative analysis of the nuclear/cytoplasmic ratio of TET1s in LCA and RCA ECs to fig.2B. All data were presented as the mean ± SD.

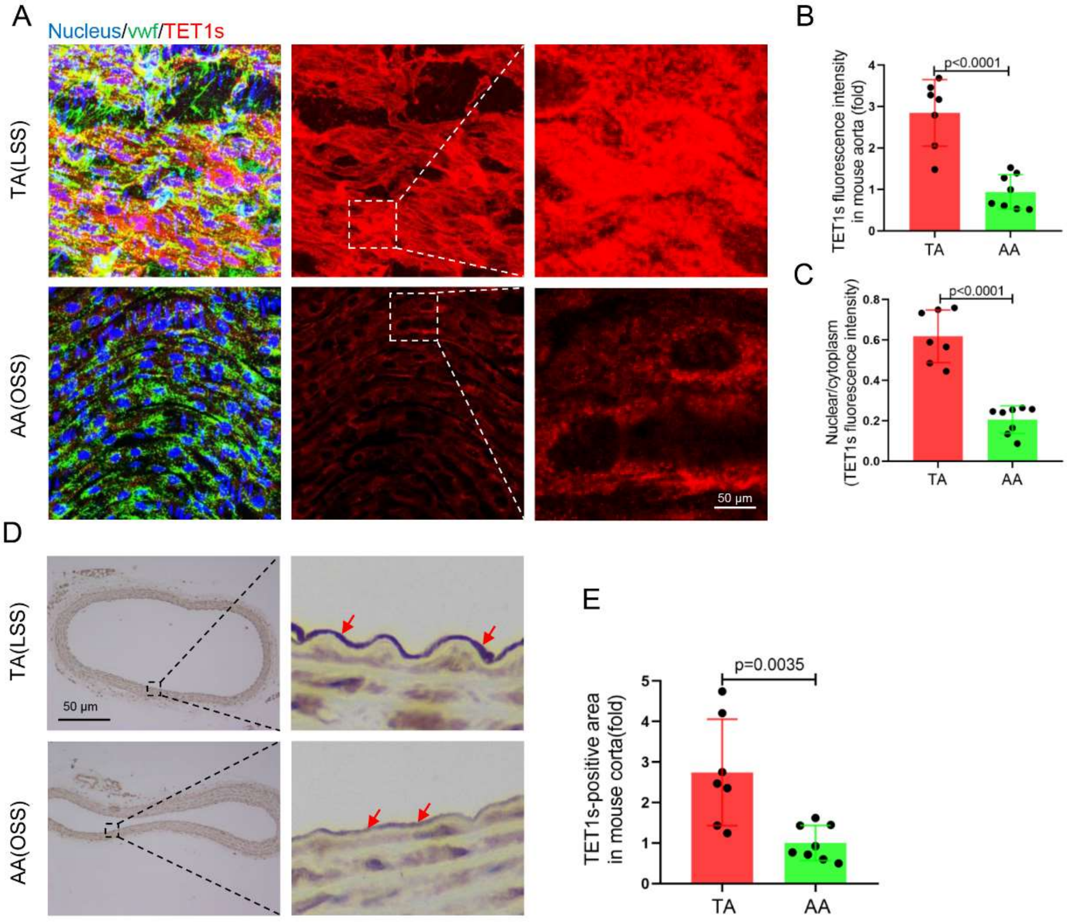

Figure S5. OSS stimulation was decreased the expression and the nuclear/cytoplasmic ratio of TET1s in aortic ECs in TET1$^{cs/cs}$ mice. (A) Immunofluorescence staining & *en face* for TET1s in AA and TA ECs (n>7 per group). (B-C) Quantitative analysis of the fluorescence intensity and the nuclear/cytoplasmic ratio of TET1s in AA and TA ECs (n>7 per group). (D-E) Immunohistochemical staining for TET1s in AA and TA slices and quantitative analysis of the TET1s-positive area; red arrows indicate the positive area in ECs (n>7 per group). All data were presented as the mean ± SD.

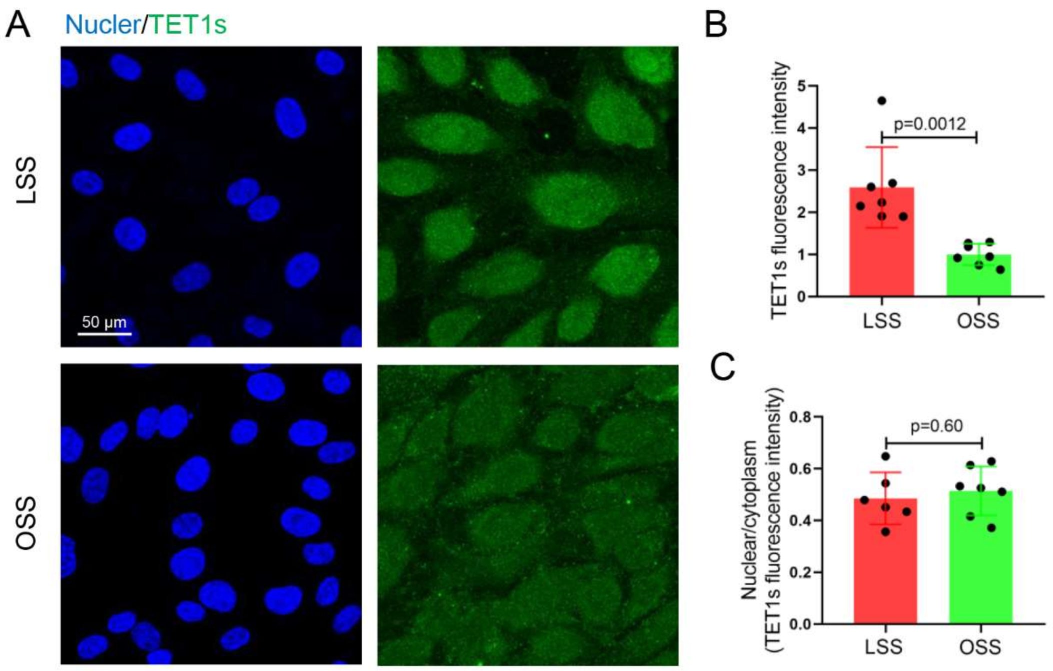

Figure S6. OSS inhibits TET1s expression levels in primary HUVECs. (A-C) primary HUVECs with a parallel-plate flow chamber (PPFC) for 24 h. (A) Immunofluorescence staining for TET1s in p-HUVECs. (B-C) Quantitative analysis of the fluorescence intensity and the nuclear/cytoplasmic ratio of TET1s in p-HUVECs (n=7 per group). All data were presented as the mean ± SD.

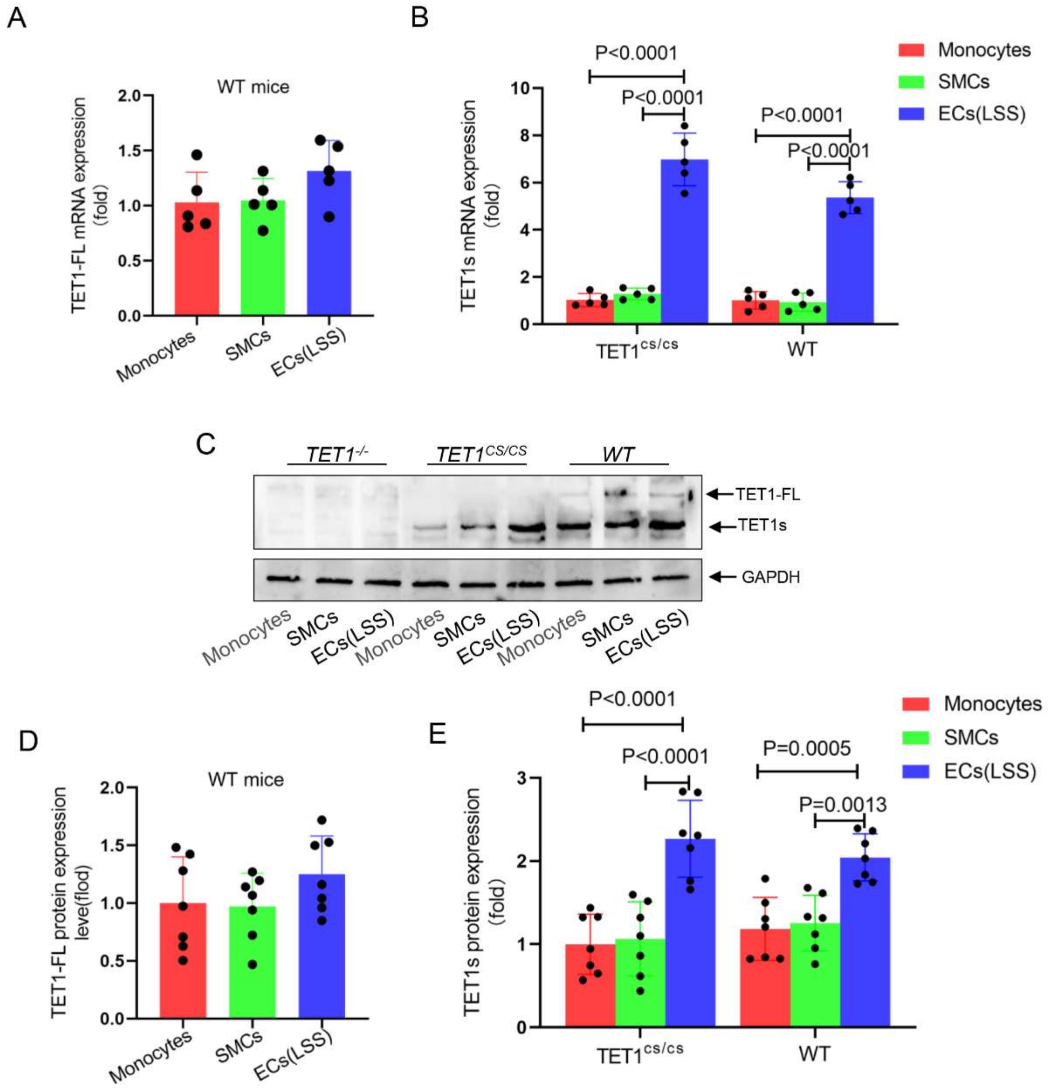

Figure S7. Low expression levels of TET1s in vascular smooth muscle cells and macrophages. (A-C) All ECs separated from the thoracic aorta (TA) of TET1$^{-/-}$, TET1$^{cs/cs}$ and WT mice. (A-B) RT-qPCR was used to test the mRNA levels of TET1s and TET1-FL (n=4 per group). (C) The TET1s and TET1-FL protein expression level was quantified by WB (n=4 per group). All data were presented as the mean ± SD.

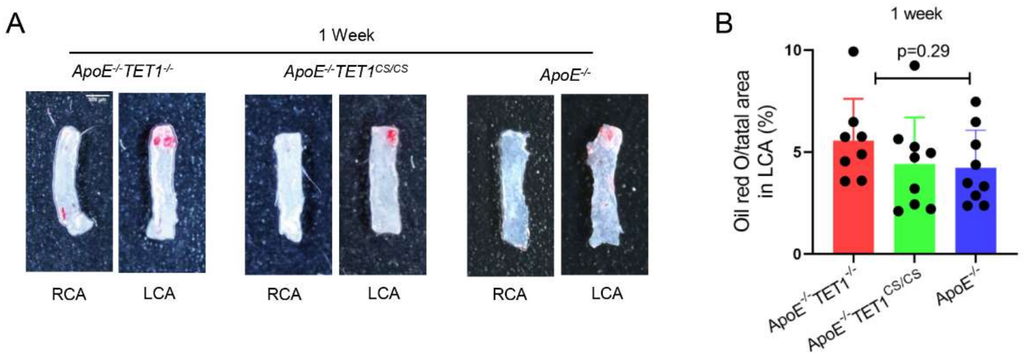

Figure S8. The carotid plaques in ApoE$^{-/-}$TET1$^{cs/cs}$ mice with a partial carotid artery ligation is no difference compared with ApoE$^{-/-}$TET1$^{-/-}$mice fed a high-fat diet for 1 week. (A-B) ApoE$^{-/-}$TET1cs/cs, ApoE$^{-/-}$ TET1$^{-/-}$ and ApoE$^{-/-}$ mice LCAs were ligated and fed a high-fat diet for 1 week; the lesion areas in the carotid artery were tested by red oil stain & en face and were analyzed (n=8 per group). All data were presented as the mean ± SD.

A

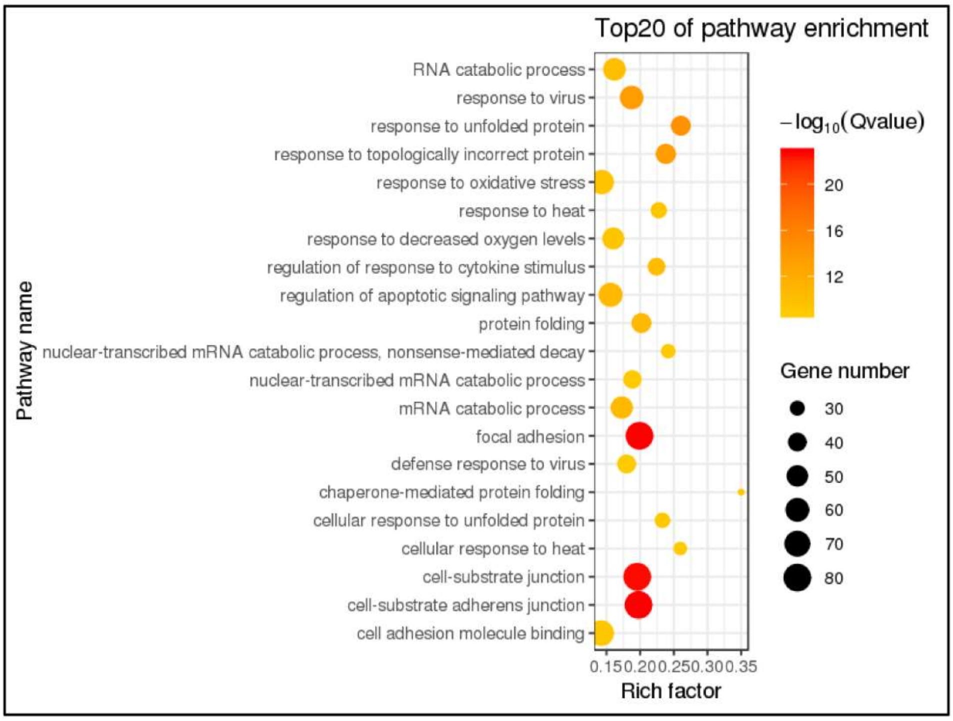

B

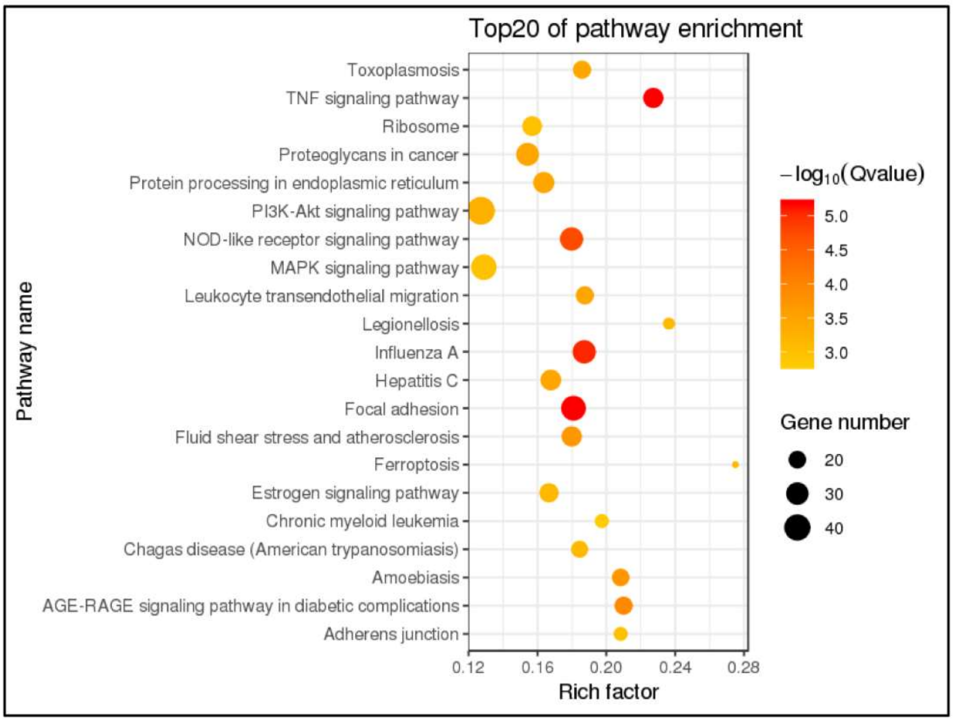

Figure S9. Differential gene expression enrichment analysis shows that TET1s may regulate endothelial barrier function. (A-B) RNA sequencing test the global RNA levels of TET1s-overexpressing p-HUVECs and negative control p-HUVECs; the top 20 pathways of differential gene expression GO enrichment analysis (A) and KEGG enrichment analysis (B) with RNA-

sequence data.

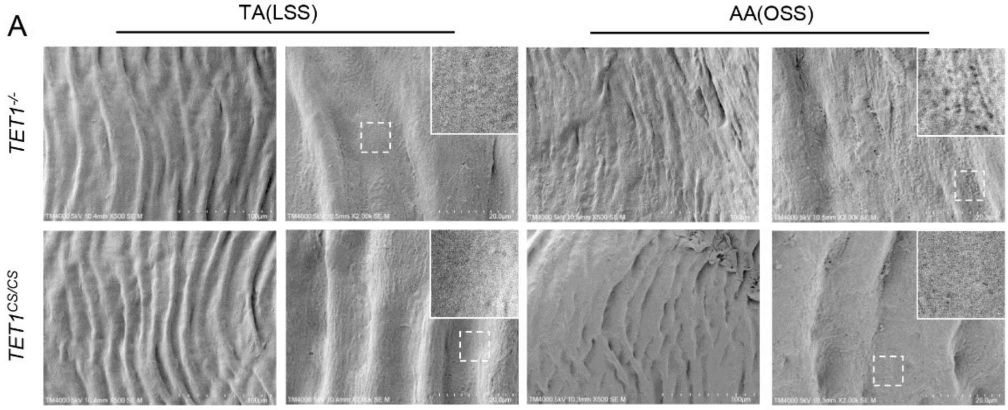

Figure S10. The intima of the aortic arch of TET1$^{cs/cs}$ mice shows fewer holes compared with TET1$^{-/-}$mice. (A) The morphology of ECs in AA and TA ECs by scanning electron microscopy (SEM).

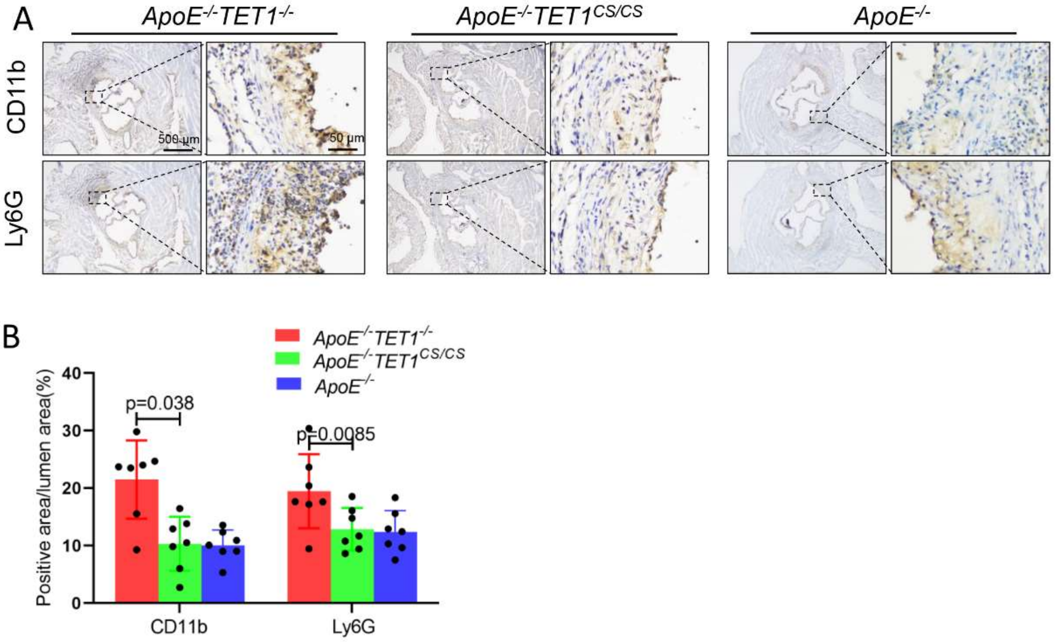

Figure S11. Neutrophils were significantly decreased in the plaques of ApoE$^{-/-}$TET1$^{cs/cs}$ mice compared with ApoE$^{-/-}$TET1$^{-/-}$ mice. (A-B) ApoE$^{-/-}$TET1cs/cs, ApoE$^{-/-}$ TET1$^{-/-}$ and ApoE$^{-/-}$ mice were fed a high-fat diet for 4 weeks. The aortic roots were harvested and subjected to further experiments. Representative immunohistochemical staining for Neutrophil-specific antigens CD11b and Ly6G in aortic roots and were analyzed (n=6 per group). All data are presented as the

mean ± SD.

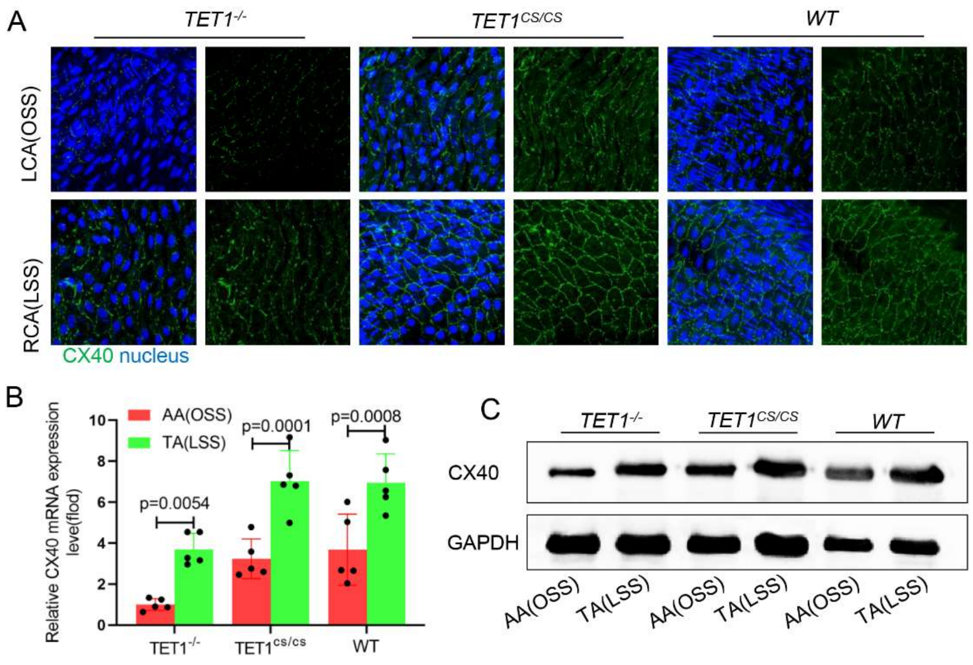

Figure S12. The endothelial CX40 expression level in TET1$^{-/-}$ and TET1$^{cs/cs}$ mice. (A) TET1$^{-/-}$, TET1$^{cs/cs}$ and WT mice LCAs were ligated for 1 week. Immunofluorescence staining for CX40 in RCA and LCA (n=3 per group). (B-C) All ECs separated from the aortic arch (AA) and thoracic aorta (TA) of TET1$^{-/-}$, TET1$^{cs/cs}$ and WT mice. (B) RT-qPCR was used to test the mRNA levels of CX40 (n=5 per group). (C-D) The CX40 protein expression level was quantified by WB (n=4 per group). All data were presented as the mean ± SD.

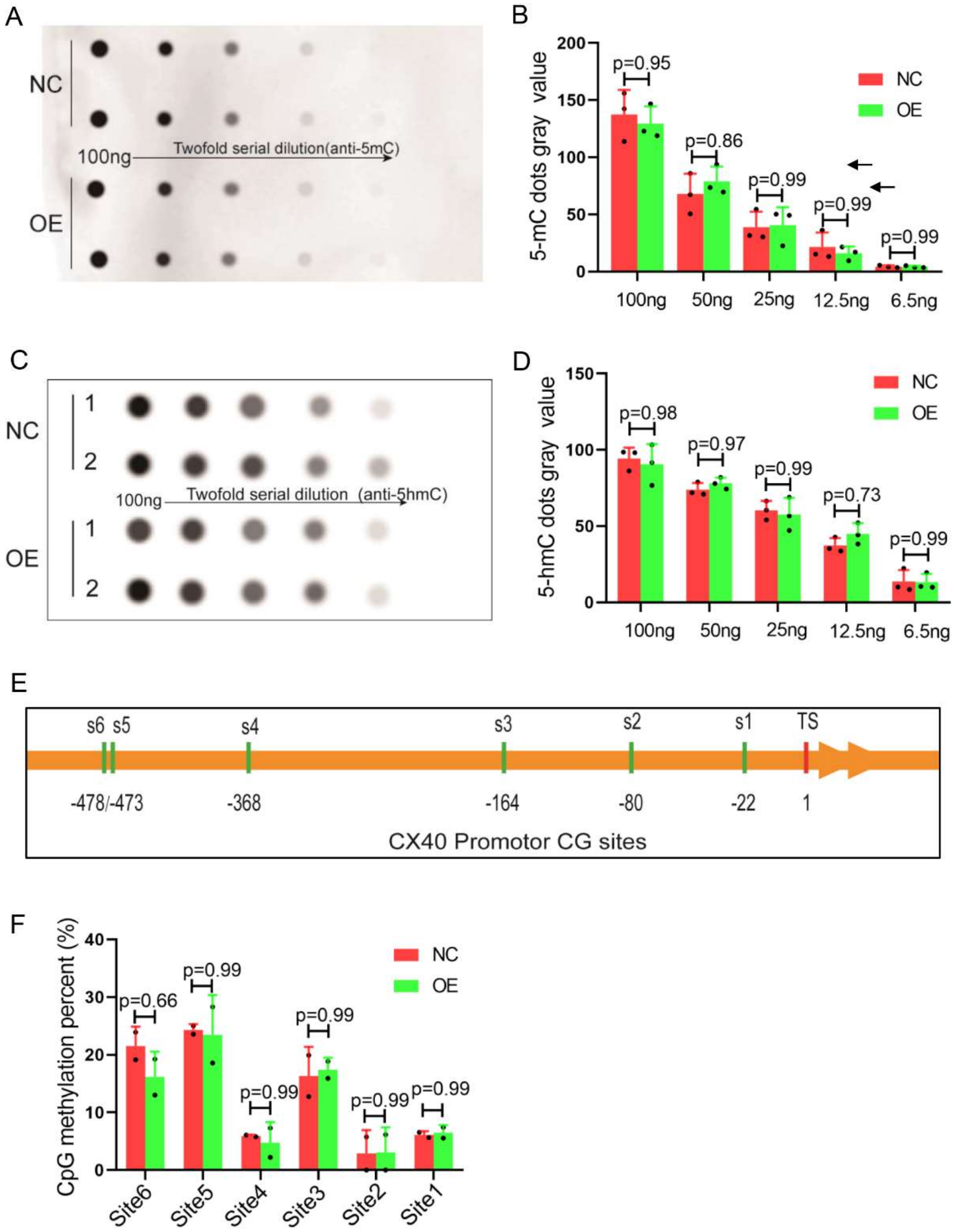

Figure S13. TET1s overexpression did not cause a global difference in 5mC levels in p-HUVECs. (A-F) p-HUVECs were transfected with TET1s-overexpressing adenovirus and negative control adenovirus and further tested after 48 h. (A-B) Dot blot assay was used to analyze the global 5mC levels of p-HUVECs (n=6 per group). (C-D) Dot blot assay was used to analyze the global 5hmC levels of p-HUVECs (n=6 per group). (E-F) Pyrosequencing assay analyzed the local 5hmC levels in the CX40 promoter 6 CG sites (S1-S6 indicates site 1-site 6; TS indicates transcriptional start; n=3 per group). All data were presented as the mean ± SD.

Table S1. The primers for gene-test of knockout mice.

primers（name）	sequence(5'-3')
GT-apoE-OF	GCCTAGCCGAGGGAGAGCCG
GT-apoE-IR	TGTGACTTGGGAGCTCTGCAGC
GT-apoE-OR	GCCGCCCCGACTGCATCT
GT-TET1-F	AACTGATTCCCTTCGTGCAG
GT-TET1-R	TTAAAGCATGGGTGGGAGTC
GT-TET1CS-OF	ATCTGGGCAATGTTGTGACTC
GT-TET1CS-IR	CATTGTAAACCCGTTGCAAGT
GT-TET1CS-OR	TTCTTTCCCTTCCACTATGCA

Table S2. The primers for RT-qPCR.

primers（name）	sequence(5'-3')
H-GAPDH-F	GGAGCGAGATCCCTCCAAAAT
H-GAPDH-R	GGCTGTTGTCATACTTCTCATGG
H-TET1FL-F	GCGCGAGTTGGAAAGTTTG
H-TET1FL-R	GCTCAGTCACACAAGGTTTTGG
H-TET1s-F	CAAGCAAGATGGCTACCTCGT
H-TET1s-R	GGGGCCTCTTGTTTTCCTTTA
H-CX40-F	GCTGCCAGAATGTCTGCTAC
H-CX40-R	GGTACTCGTAAGAGCCAGAGC
H-TMEM129-F	GAGGTGACCTTCACTCTCGC
H-TMEM129-R	GCCCACATAGTAGCCGAGC
H-TGFA-F	AGGTCCGAAAACACTGTGAGT
H-TGFA-R	AGCAAGCGGTTCTTCCCTTC
H-IGF1-F	GCTCTTCAGTTCGTGTGTGGA

H-IGF1-R	GCCTCCTTAGATCACAGCTCC
H-APLNR-F	CCTGCATCAGCTACGTCAACA
H-APLNR-R	GGGATGGATTTCTCGTGCATCT
H-PIEZO2-F	ATGGCCTCAGAAGTGGTGTG
H-PIEZO2-F	ATGTCCTTGCATCGTCGTTTT
H-KLF2-F	CTACACCAAGAGTTCGCATCTG
H-KLF2-R	CCGTGTGCTTTCGGTAGTG
H-KLF4-F	CAGCTTCACCTATCCGATCCG
H-KLF4-R	GACTCCCTGCCATAGAGGAGG
M-TET1FL-R	TACTGCAAGAATCGAAAGAACAGCCA
M-TET1FL-R	CGGAAGGTGTGTGTCAGTGGGT
M-TET1s-F	TAAGACAGACTTTTAGGGGGAAAG
M-TET1s-R	GTGTGTGTCAGTGGGTAAACAGT
M-GAPDH-F	TGACCTCAACTACATGGTCTACA
M-GAPDH-R	CTTCCCATTCTCGGCCTTG
M-CX40-F	GGTCCACAAGCACTCCACAG
M-CX40-R	CTGAATGGTATCGCACCGGAA

Table S3. The primers for CHIP- qPCR.

primers（name）	sequence(5’-3’)
P1-F	TCCTGTCACTGAGGAAATTCCTGTTC
P1-R	TGCTGTCTGAGATGGCTCTTAATGAG
P2-F	TCATTAAGAGCCATCTCAGACAGCAG
P2-R	AATCTCTGATGCTGGCCTTGC
P3-F	AAGGCAAGGCCAGCATCAG
P3-R	TCCTGTGCATGACTTTCTGGAATG
P4-F	CTCATTCCAGAAAGTCATGCACAGG
P4-R	GCCTGAAGTCAAGCTTGTCTGG
P5-F	CCAGACAAGCTTGACTTCAGGC

P5-R	GAGATCTTGTCCTGAGAGCATTATGCTC

Table S4. The primers for pyrosequencing assays.

primers（name）	sequence(5’-3’)
1.GJA5-1F(230bp)	TTTGGGTGAAAGTTTTATTTGGATATG
1.GJA5-1R	TCCCAACTTTAATCTACTCCTATCACT
1.GJA5-1S	CTCAAAAACATTTAACTTCC
2.GJA5-2F(72bp)	TGGTTTTTTGGGTGAAAGTTT
2.GJA5-2R	AAACCATCTCAAACAACAAATATCTATTAA
2.GJA5-2S	TTTTGGGTGAAAGTTTTATT
3.GJA5-3F(224bp)	AGAGGATTAGAAAAGGTAAGGTTAGTAT
3.GJA5-3R	CCTCTTTAAAACCTAAAATCAAACTTATCT
3.GJA5-3S	AGAGGTTTTTAAGTAAATAGTG
4.GJA5-2F(221bp)	AAGGAGATTTTGTTTTGAGAGTATTATG
4.GJA5-4R	AACAATACTACCCATCCTTTCAACTACCC
4.GJA5-4S	ATTAAAAAGGAAGTTAGATTGT
5.GJA5-5F(140bp)	TGATTTTAGGTTTTAAAGAGGAAGTTAATG
5.GJA5-5R	ACTCAACCCTTCCCTAAC
5.GJA5-5S	ACCCTTCCCTAACTC
